# Supplementary figures and images for: The suprachiasmatic nucleus regulates brown fat thermogenesis in male mice through an adrenergic receptor ADRB3-S100B signaling pathway
Source: PLoS Biol. 2025 Dec 4;23(12):e3003534. doi: 10.1371/journal.pbio.3003534 (PMC12688110; doi:10.1371/journal.pbio.3003534)

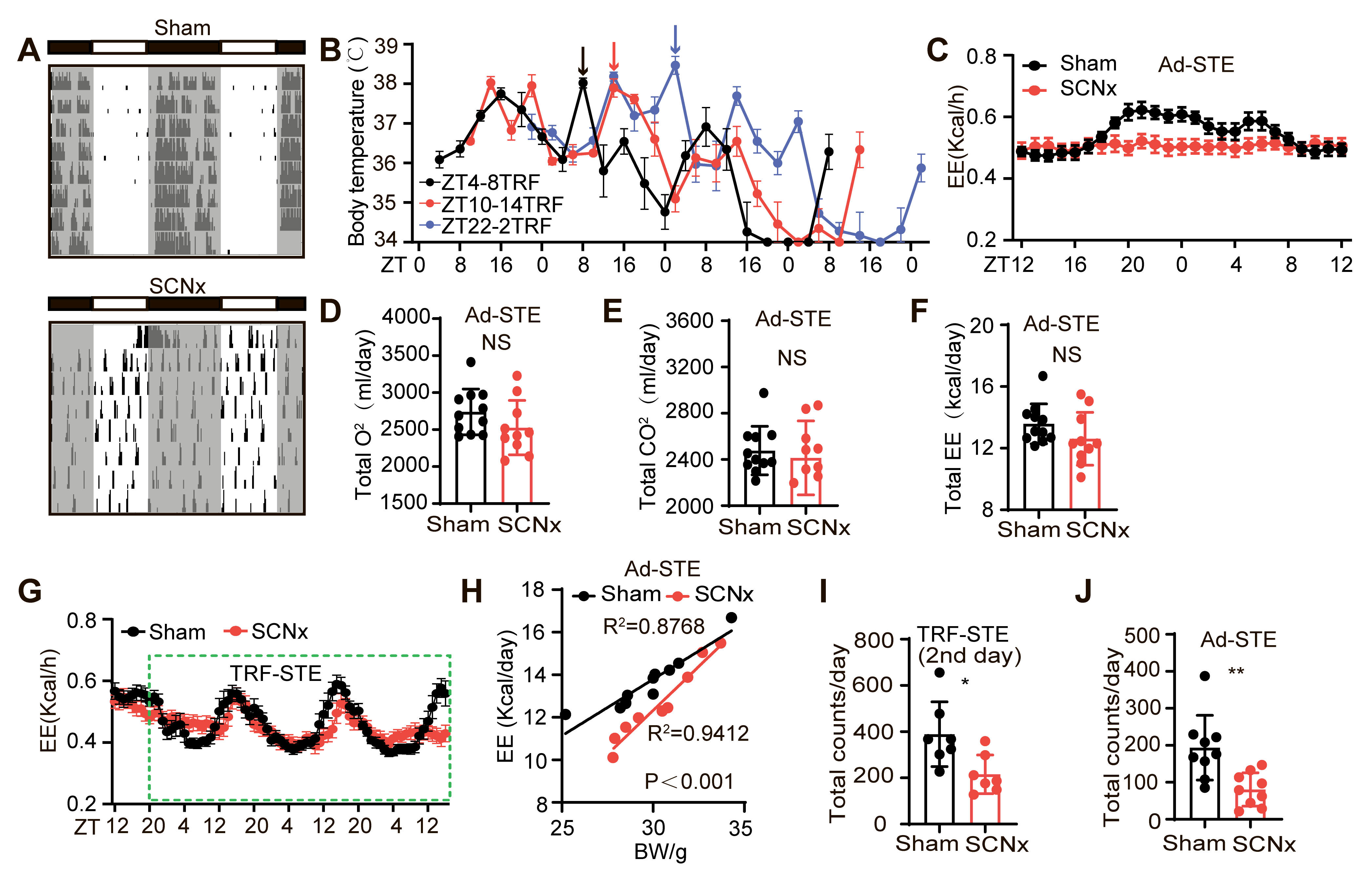

Supplement: S1 Fig — (A) Representative actograms showing locomotor activity patterns in sham (top) and SCNx (bottom) mice used for screening of behavioral arrhythmicity following SCN lesioning. (B) CBT profiles in WT mice subjected to TRF-STE initiated at different circadian windows (ZT4-8, ZT10-14, and ZT22-2). Arrows indicate the time of TRF onset. Data are reanalyzed from Zhang and colleagues, 2020 and presented as mean ± SEM (n = 4 per group). (C) Representative 24-hour plot of EE measured by indirect calorimetry in sham and SCNx mice under Ad-STE. Data shown as mean ± SEM (sham: n = 11; SCNx: n = 10). (D–F) Total O₂ consumption (D), CO₂ production (E), and EE (F) during Ad-STE. Data presented as mean ± SEM (sham: n = 11; SCNx: n = 10). (G) Three-day EE profiles under TRF-STE measured by indirect calorimetry. Data presented as mean ± SEM (n = 10 per group). (H) GLM analysis of EE under TRF-STE, using group (sham versus SCNx) as the independent variable and body weight as a covariate. Sham: n = 11; SCNx: n = 10. (I and J) Statistical analysis of total physical activity from the second day under TRF-STE (I, n = 7) and Ad-STE (J, n = 9) as recorded in metabolic cages. Except as otherwise indicated, data are presented as mean ± SD. NS: not significant, *p < 0.05 and **p < 0.01 as determined by unpaired two-tailed Student t test (D–F, I, and J). The data underlying the graphs shown in the figure can be found in S1 Source Data. (TIF) [file pbio.3003534.s001.tif]

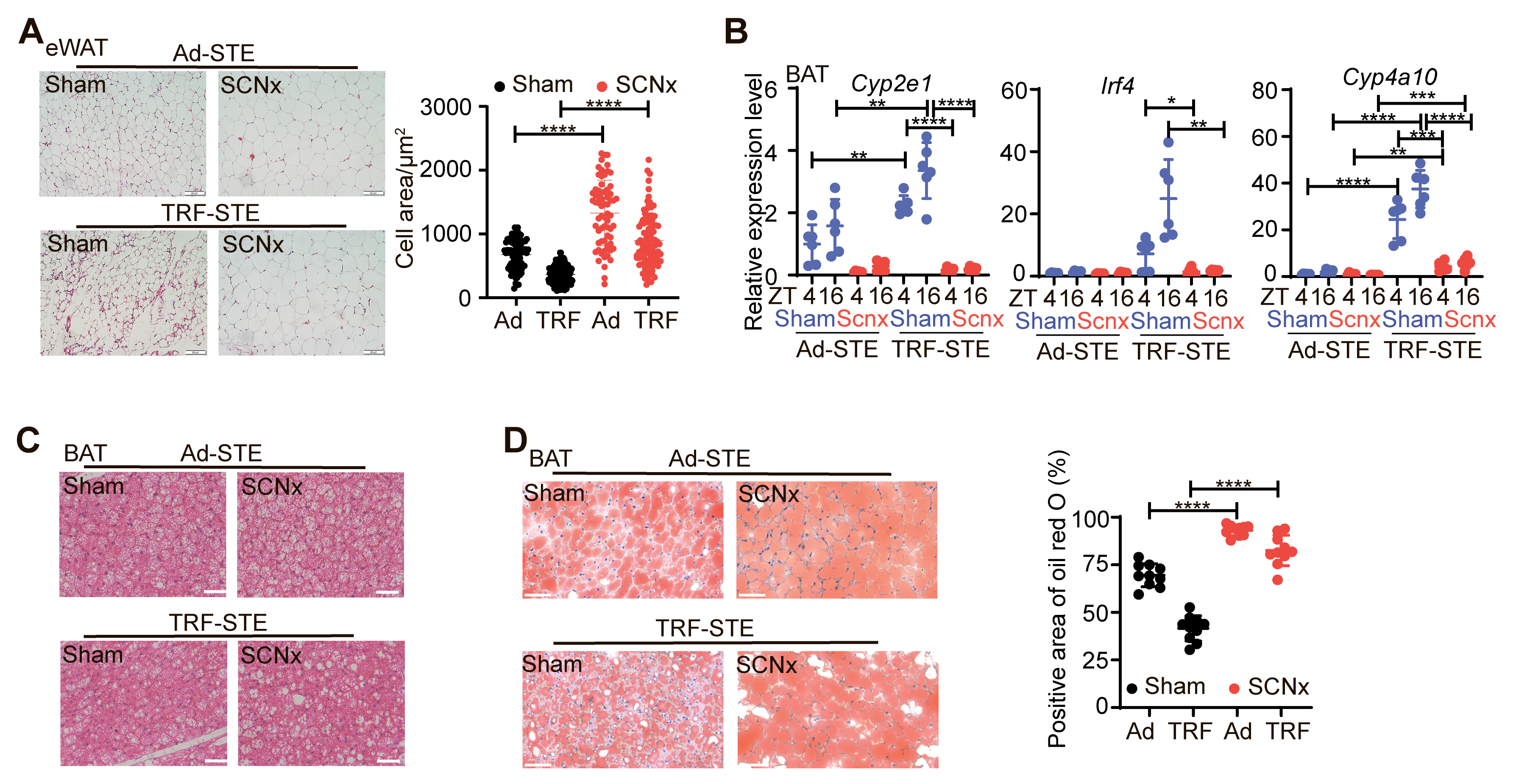

Supplement: S2 Fig — (A) Representative H&E-stained images of eWAT collected at ZT16 under Ad-STE and TRF-STE conditions (left), with quantification of adipocyte cross-sectional areas (right). Scale bars, 50 μm. (B) mRNA expression of genes involved in fatty acid mobilization and oxidation in interscapular BAT from sham and SCNx mice under Ad-STE and TRF-STE (n = 6 per group). (C) Representative H&E-stained sections of interscapular BAT collected at ZT16 under Ad-STE and TRF-STE conditions. Scale bars, 50 μm. (D) Representative Oil Red O-stained sections of interscapular BAT collected at ZT16 under Ad-STE and TRF-STE (left), with quantification of the Oil Red O-positive area (right). Scale bars, 50 μm. Data are presented as mean ± SD. *p < 0.05, **p < 0.01, ***p < 0.001, and ****p < 0.0001 as determined by unpaired two-tailed Student t test (A, B, and D). The data underlying the graphs shown in the figure can be found in S1 Source Data. (TIF) [file pbio.3003534.s002.tif]

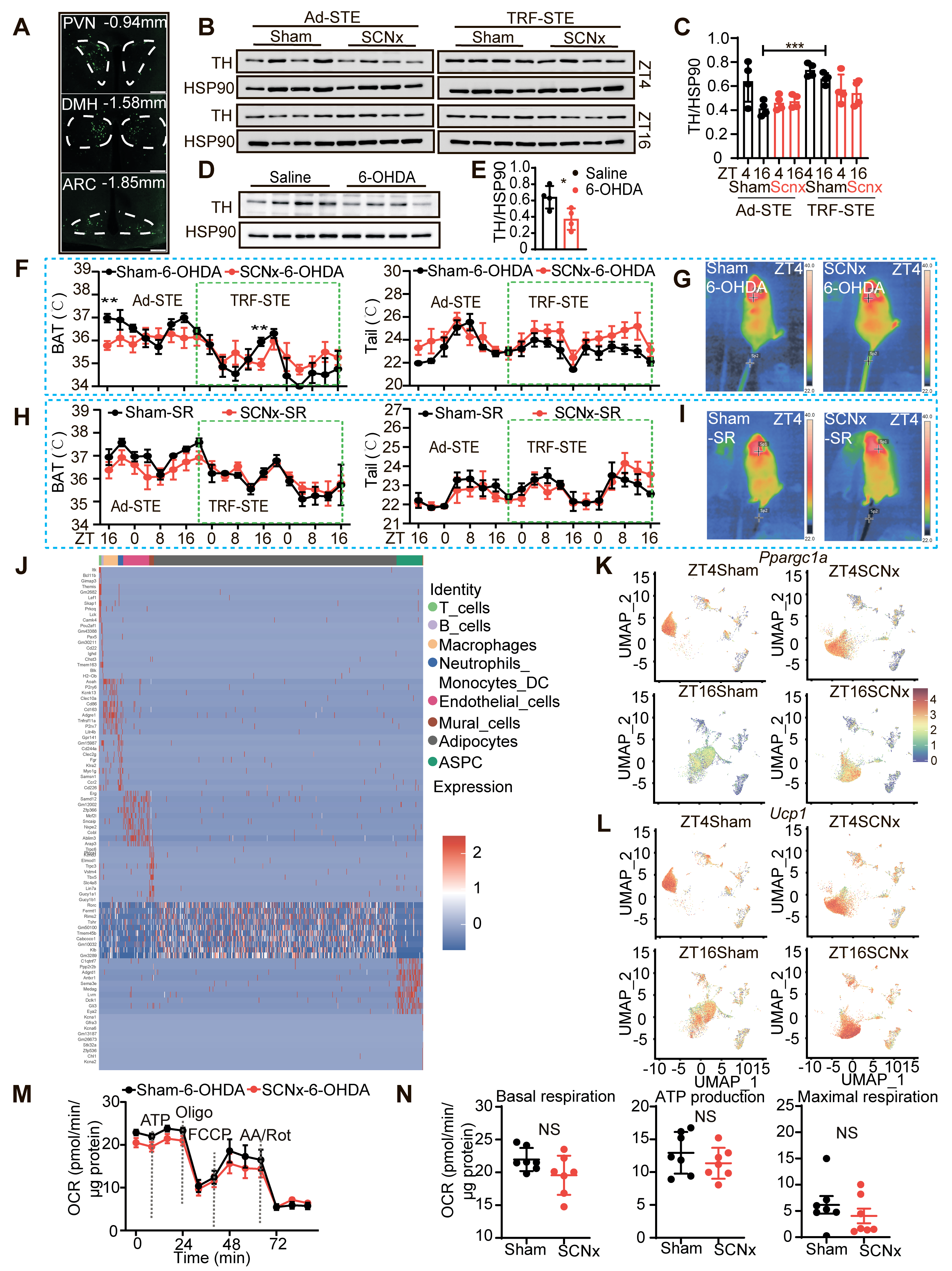

Supplement: S3 Fig — (A) Retrograde tracing showing terminal projections in the PVN, DMH, and ARC regions. Scale bar, 100 μm. (B and C) Western blots analysis of TH in interscapular BAT (B) from sham and SCNx mice at ZT4 and ZT16 under Ad-STE and TRF-STE, with densitometry analysis (C). n = 4 per group. (D and E) Western blots analysis of TH (D) in interscapular BAT from 6-OHDA-treated mice, with densitometry analysis (E). n = 4 per group. (F and G) Changes in interscapular BAT and tail temperatures in response to 6-OHDA treatment in sham and SCNx mice under Ad-STE to TRF-STE (F). Data presented as mean ± SEM, n = 6 per group. Representative thermographic images illustrate body surface temperature (G). (H and I) Changes in interscapular BAT and tail temperatures following SR59230A treatment in sham and SCNx mice under Ad-STE to TRF-STE (H). Data presented as mean ± SEM, sham-SR mice n = 6, SCNx-SR mice n = 5. Representative thermographic images illustrate body surface temperature (I). SR: SR59230A. (J) SnRNA-seq analysis identified eight major cell clusters (S1 Table). (K and L) UMAP plots showing Log2 expression levels of Pgc1α (K) and Ucp1 (L) in sham and SCNx mice at ZT4 and ZT16 under TRF-STE. (M and N) Mitochondrial OCR measurements using Seahorse XF Analyzer in interscapular BAT mitochondria isolated from 6-OHDA-treated sham and SCNx mice (M), with quantification of basal respiration, ATP-linked respiration, and maximal respiration (N). n = 7 per group. Unless otherwise indicated, data are presented as mean ± SD. NS: not significant, *p < 0.05, **p < 0.01, and ***p < 0.01, unpaired two-tailed Student t test (C, E, and N), two-way ANOVA with Sidak’S multiple comparisons test (F and H). The data underlying the graphs shown in the figure can be found in S1 Source Data. Raw blot images can be found in S1 Raw Images. (TIF) [file pbio.3003534.s003.tif]

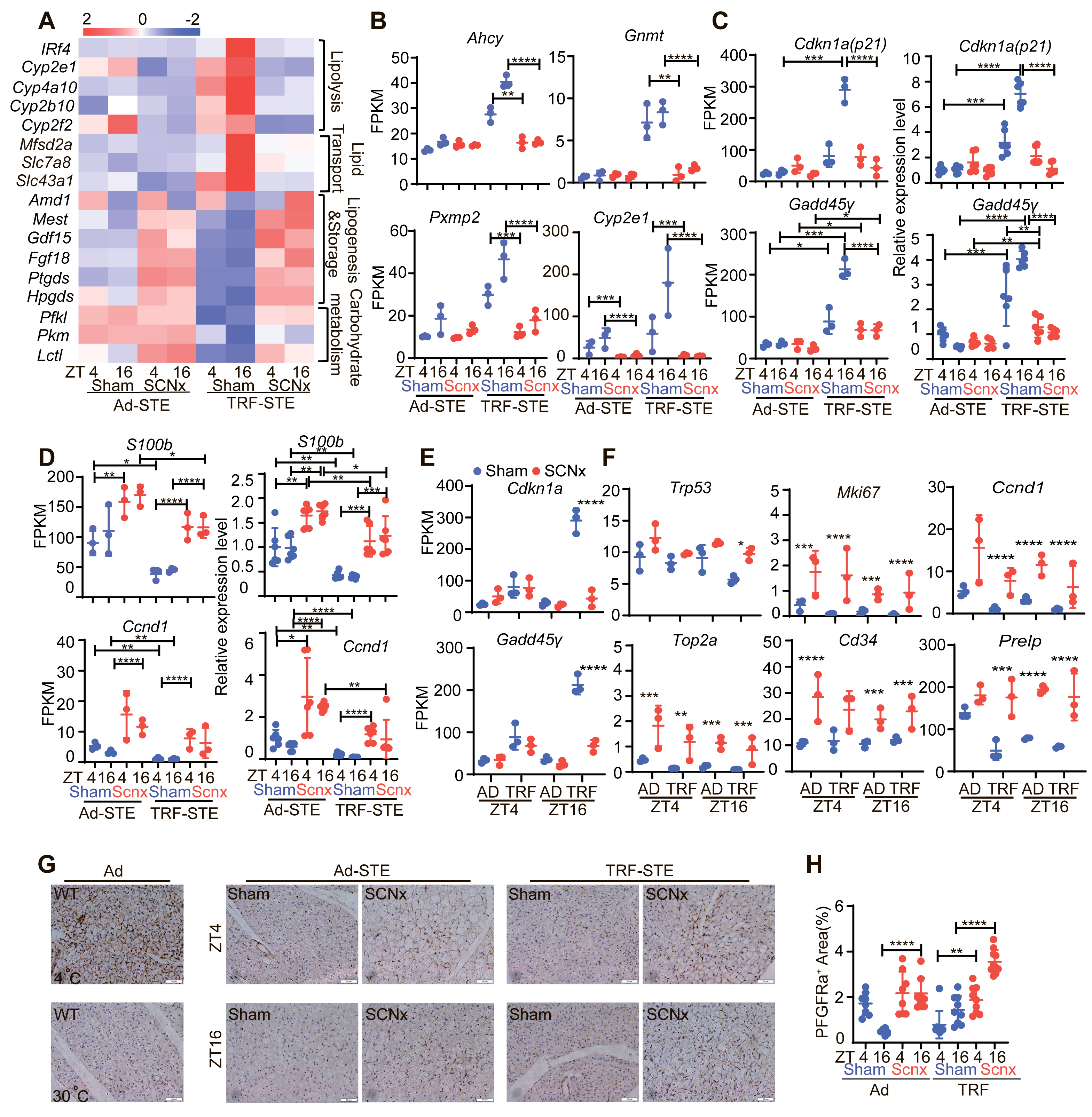

Supplement: S4 Fig — (A) A heatmap based on RNA-seq analysis of genes related to lipolysis, lipid transport, lipogenesis, and carbohydrate metabolism. (B) Gene expression levels involved in methylation and detoxification based on RNA-seq analysis. (fold change > 2, p < 0.01). n = 3 per group. (C) Validation of senescence markers (Cdkn1a, Gadd45γ) using qPCR in sham and SCNx interscapular BAT under Ad-STE and TRF-STE at ZT4 and ZT16. Left: RNA-seq expression values. n = 3 per group; right: corresponding qPCR. n = 6 per group. (D) Validation of proliferation-related genes (S100b, Ccnd1) by qPCR under the same conditions as in (C). Left: RNA-seq expression values. n = 3 per group; right: corresponding qPCR. n = 6 per group. (E and F) Genes significantly altered in the interscapular BAT of SCNx mice at both ZT4 and ZT16 under Ad-STE and TRF-STE (fold change > 2, p < 0.01). n = 3 per group. (G and H) Representative images of anti-PDGFRα immunostaining in interscapular BAT sections from sham and SCNx mice under Ad-STE and TRF-STE conditions, with 4°C and 30°C acclimation included as thermal controls (G). Quantification of PDGFRα-positive cells is shown in (H). Scale bars, 50 μm. Data are presented as mean ± SD. *p < 0.05, **p < 0.01, ***p < 0.001, and ***p < 0.0001 as determined by unpaired two-tailed Student t test (B-F, H). The data underlying the graphs shown in the figure can be found in S1 Source Data. (TIF) [file pbio.3003534.s004.tif]

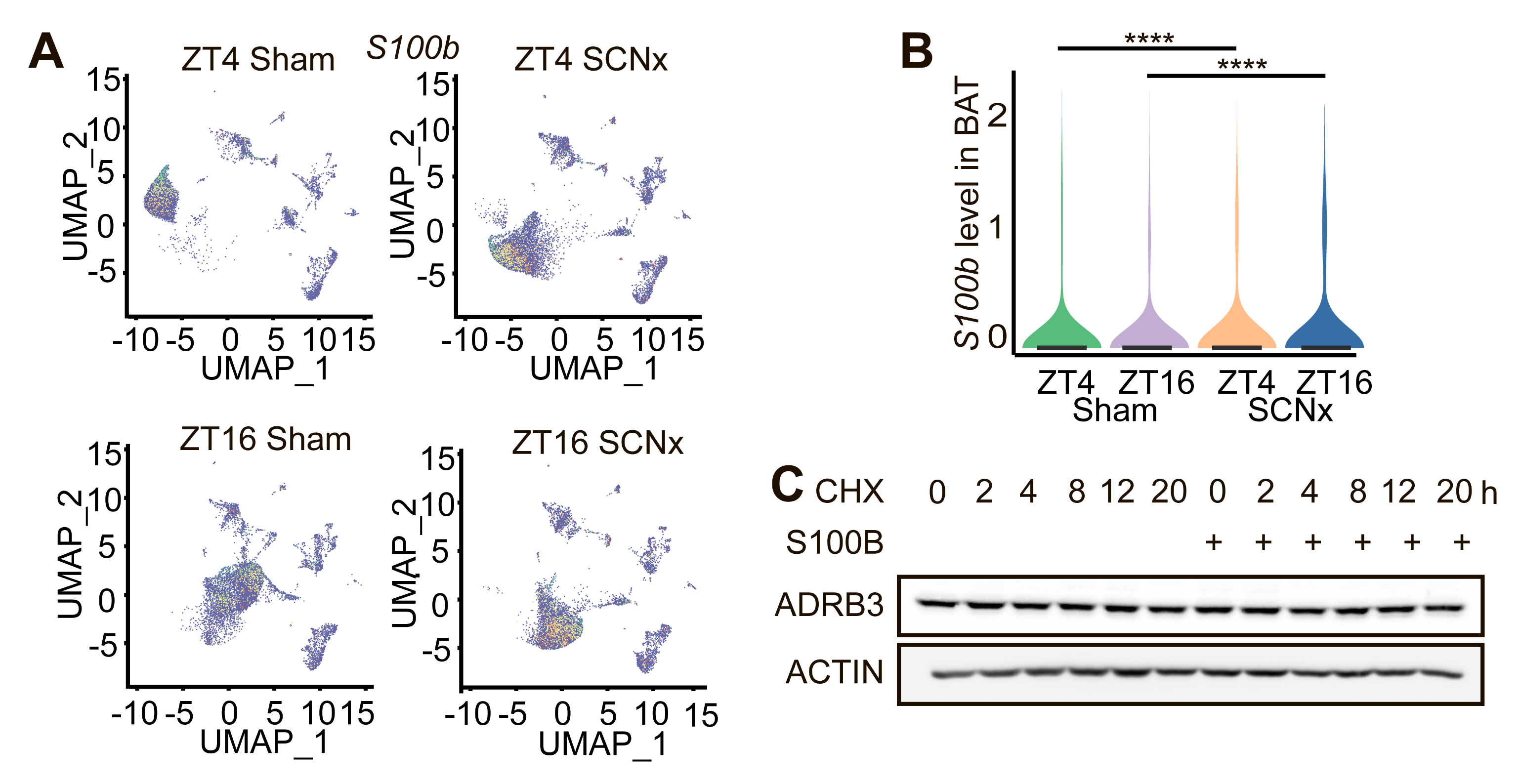

Supplement: S5 Fig — (A) UMAP plots display Log2 expression levels of S100b in sham, and SCNx mice at ZT4 and ZT16 under TRF-STE. (B) Violin plots depicting log2 expression levels of S100b in adipocytes from sham and SCNx mice at ZT4 and ZT16 under TRF-STE. (C) Immunoblot analysis of ADRB3 stability in 293T cells overexpressing Adrb3 and treated with recombinant S100B protein and cycloheximide (CHX). The assay was performed to evaluate the effect of S100B on ADRB3 protein stability. Raw blot images can be found in S1 Raw Images. (TIF) [file pbio.3003534.s005.tif]

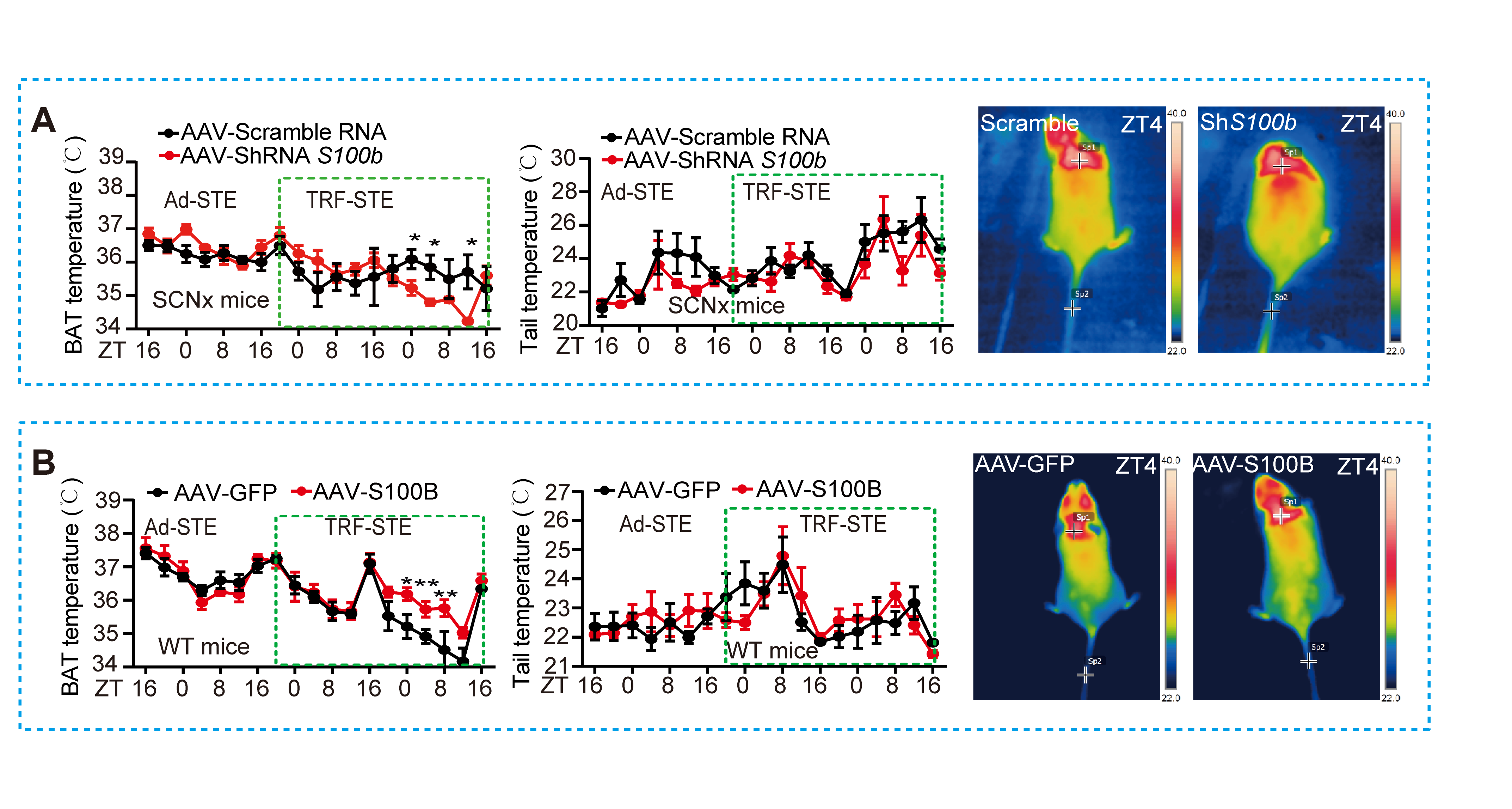

Supplement: S6 Fig — (A) Interscapular BAT and tail temperature as indicated in responses to scramble or knockdown S100b in SCNx mice from Ad-STE to TRF-STE. ShRNA S100b mice n = 5, scramble RNA mice n = 6. Representative thermographic images illustrate body surface temperature. (B) Interscapular BAT and tail temperature as indicated in responses to EGFP or overexpressed S100b in WT mice from Ad-STE to TRF-STE, n = 6 per group. Representative thermographic images illustrate body surface temperature. Data presented as mean ± SEM, *p < 0.05 and **p < 0.01, two-way ANOVA with Sidak’S multiple comparisons test (A, B). The data underlying the graphs shown in the figure can be found in S1 Source Data. (TIF) [file pbio.3003534.s006.tif]

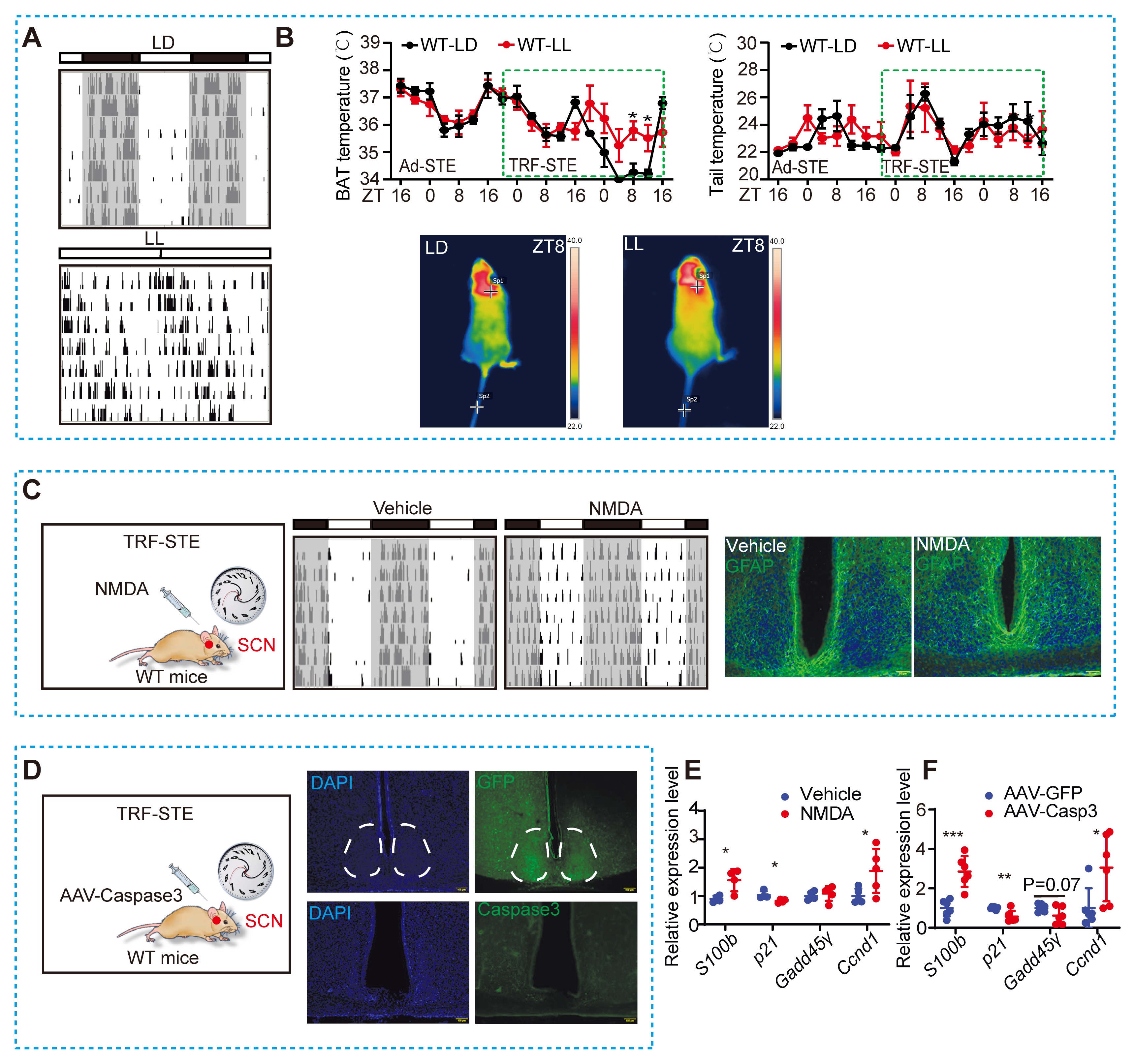

Supplement: S7 Fig — (A) Locomotor screening for arrhythmic activity in mice following constant light treatment for 2 months. (B) Interscapular BAT and tail temperature responses in WT mice under LD or LL conditions from Ad-STE to TRF-STE. Data presented as mean ± SEM, n = 4 per group. Representative thermographic images illustrate body surface temperature. (C) Schematic of NMDA treatment (left), locomotor screening (middle), and representative immunofluorescence images of GFAP staining in SCN (right). n = 5 per group. (D) Validation of AAV-DIO-Casp3-GFP and AAV-VGAT1-Cre injection in the SCN. n = 6 per group. (E and F) Relative mRNA expression of the indicated genes after NMDA (E) or Caspase-3 injecting treatment (F). Except as otherwise indicated, data are presented as mean ± SD. *p < 0.05, **p < 0.01, and ***p < 0.001, unpaired two-tailed Student t test (E and F), two-way ANOVA with Sidak’S multiple comparisons test (B). Scale bars, 50 μm, (C) 100 μm (D). Schematic (C, D) created in BioRender.com. The data underlying the graphs shown in the figure can be found in S1 Source Data. (TIF) [file pbio.3003534.s007.tif]
