# Supplementary material for: The suprachiasmatic nucleus regulates brown fat thermogenesis in male mice through an adrenergic receptor ADRB3-S100B signaling pathway
Source: PLoS Biol. 2025 Dec 4;23(12):e3003534. doi: 10.1371/journal.pbio.3003534 (PMC12688110; doi:10.1371/journal.pbio.3003534)
Supplement: S1 Table — (DOCX) [file pbio.3003534.s008.docx]

**S1 Table.** **The top 10 markers for each cluster in snRNA-seq.**

| Gene | avg_log2FC | pct.1 | pct.2 | Cluster | Gene diff |
| --- | --- | --- | --- | --- | --- |
| *Tmem45b* | 0.898562651 | 0.334 | 0.115 | Adipocytes | 2.904 |
| *Gm3289* | 0.857873527 | 0.444 | 0.156 | Adipocytes | 2.846 |
| *Klb* | 0.80316024 | 0.407 | 0.142 | Adipocytes | 2.866 |
| *Cabcoco1* | 0.775025614 | 0.355 | 0.123 | Adipocytes | 2.886 |
| *Rims2* | 0.763919068 | 0.312 | 0.104 | Adipocytes | 3 |
| *Rorc* | 0.734068918 | 0.338 | 0.109 | Adipocytes | 3.101 |
| *Tshr* | 0.726280507 | 0.254 | 0.086 | Adipocytes | 2.953 |
| *Gm50100* | 0.668607126 | 0.339 | 0.116 | Adipocytes | 2.922 |
| *Gm10032* | 0.656901965 | 0.314 | 0.11 | Adipocytes | 2.855 |
| *Fermt1* | 0.608947721 | 0.258 | 0.086 | Adipocytes | 3 |
| *Lvrn* | 2.85352876 | 0.54 | 0.015 | ASPC | 36 |
| *Antxr1* | 2.635337732 | 0.54 | 0.014 | ASPC | 38.571 |
| *Adgrd1* | 2.541952673 | 0.492 | 0.01 | ASPC | 49.2 |
| *Sema3e* | 2.071684019 | 0.268 | 0.007 | ASPC | 38.286 |
| *Ppp2r2b* | 1.970299619 | 0.271 | 0.005 | ASPC | 54.2 |
| *C1qtnf7* | 1.936157478 | 0.312 | 0.005 | ASPC | 62.4 |
| *Gli3* | 1.862149941 | 0.384 | 0.011 | ASPC | 34.909 |
| *Dclk1* | 1.858536612 | 0.319 | 0.009 | ASPC | 35.444 |
| *Medag* | 1.756970689 | 0.342 | 0.009 | ASPC | 38 |
| *Eya2* | 1.582377251 | 0.275 | 0.008 | ASPC | 34.375 |
| *Pax5* | 3.087457329 | 0.659 | 0.002 | B cells | 329.5 |
| *Tmem163* | 2.976096664 | 0.546 | 0.003 | B cells | 182 |
| *Ighd* | 2.445008088 | 0.491 | 0.002 | B cells | 245.5 |
| *Gm43388* | 2.378734786 | 0.406 | 0.001 | B cells | 406 |
| *Chst3* | 2.029064175 | 0.369 | 0.002 | B cells | 184.5 |
| *Blk* | 1.869572689 | 0.307 | 0.002 | B cells | 153.5 |
| *Gm30211* | 1.809309307 | 0.307 | 0.001 | B cells | 307 |
| *H2-Ob* | 1.791681858 | 0.294 | 0.002 | B cells | 147 |
| *Pou2af1* | 1.492191521 | 0.266 | 0 | B cells | Inf |
| *Cd22* | 1.481097165 | 0.256 | 0.001 | B cells | 256 |
| *Ablim3* | 2.92987986 | 0.626 | 0.022 | Endothelial cells | 28.455 |
| *Samd12* | 2.407052612 | 0.389 | 0.011 | Endothelial cells | 35.364 |
| *Mcf2l* | 2.054103903 | 0.423 | 0.013 | Endothelial cells | 32.538 |
| *Gm12002* | 1.922508106 | 0.347 | 0.01 | Endothelial cells | 34.7 |
| *Nxpe2* | 1.72018438 | 0.323 | 0.011 | Endothelial cells | 29.364 |
| *Sncaip* | 1.700278524 | 0.305 | 0.01 | Endothelial cells | 30.5 |
| *Zfp366* | 1.651379689 | 0.297 | 0.009 | Endothelial cells | 33 |
| *Cobl* | 1.402362748 | 0.26 | 0.009 | Endothelial cells | 28.889 |
| *Erg* | 1.368569427 | 0.252 | 0.006 | Endothelial cells | 42 |
| *Arap3* | 1.34739168 | 0.256 | 0.009 | Endothelial cells | 28.444 |
| *Cd163* | 2.677144936 | 0.469 | 0.012 | Macrophages | 39.083 |
| *Aoah* | 2.475423223 | 0.478 | 0.009 | Macrophages | 53.111 |
| *Adgre1* | 2.313123043 | 0.52 | 0.015 | Macrophages | 34.667 |
| *Kcnk13* | 1.980631432 | 0.384 | 0.009 | Macrophages | 42.667 |
| *Lilr4b* | 1.791335424 | 0.304 | 0.009 | Macrophages | 33.778 |
| *Cd86* | 1.669666203 | 0.284 | 0.007 | Macrophages | 40.571 |
| *Clec10a* | 1.525026039 | 0.289 | 0.007 | Macrophages | 41.286 |
| *Tnfrsf11a* | 1.417083927 | 0.275 | 0.008 | Macrophages | 34.375 |
| *P2rx7* | 1.416526952 | 0.273 | 0.008 | Macrophages | 34.125 |
| *P2ry6* | 1.274880383 | 0.253 | 0.005 | Macrophages | 50.6 |
| *Trpc3* | 2.724555774 | 0.666 | 0.004 | Mural_cells | 166.5 |
| *Trpc6* | 2.646202883 | 0.569 | 0.002 | Mural_cells | 284.5 |
| *Lin7a* | 2.548144528 | 0.585 | 0.005 | Mural_cells | 117 |
| *Kcnd3* | 2.155321245 | 0.456 | 0.002 | Mural_cells | 228 |
| *Gucy1a1* | 2.003071631 | 0.455 | 0.004 | Mural_cells | 113.75 |
| *Tbx5* | 1.964457078 | 0.433 | 0.003 | Mural_cells | 144.333 |
| *Gucy1b1* | 1.758937985 | 0.399 | 0.004 | Mural_cells | 99.75 |
| *Elmod1* | 1.6647927 | 0.349 | 0.002 | Mural_cells | 174.5 |
| *Vstm4* | 1.357190976 | 0.301 | 0.002 | Mural_cells | 150.5 |
| *Slc4a8* | 1.289719578 | 0.284 | 0.002 | Mural_cells | 142 |
| *Ccr2* | 2.639235469 | 0.523 | 0.013 | Neutrophils Monocytes DC | 40.231 |
| *Fgr* | 2.593818539 | 0.466 | 0.008 | Neutrophils Monocytes DC | 58.25 |
| *Gm15987* | 2.32086114 | 0.496 | 0.005 | Neutrophils Monocytes DC | 99.2 |
| *Gpr141* | 2.191004292 | 0.319 | 0.002 | Neutrophils Monocytes DC | 159.5 |
| *Cd226* | 2.17884972 | 0.357 | 0.009 | Neutrophils Monocytes DC | 39.667 |
| *Samsn1* | 2.115842029 | 0.359 | 0.008 | Neutrophils Monocytes DC | 44.875 |
| *Klra2* | 1.923010386 | 0.273 | 0.006 | Neutrophils Monocytes DC | 45.5 |
| *Myo1g* | 1.857670001 | 0.407 | 0.009 | Neutrophils Monocytes DC | 45.222 |
| *Cd244a* | 1.76191426 | 0.274 | 0.003 | Neutrophils Monocytes DC | 91.333 |
| *Clec2g* | 1.648384834 | 0.334 | 0.004 | Neutrophils Monocytes DC | 83.5 |
| *Gm2682* | 4.762810401 | 0.817 | 0.003 | T cells | 272.333 |
| *Skap1* | 4.438070405 | 0.903 | 0.005 | T cells | 180.6 |
| *Lef1* | 3.373955527 | 0.488 | 0.002 | T cells | 244 |
| *Themis* | 3.266142011 | 0.584 | 0.002 | T cells | 292 |
| *Prkcq* | 3.077885423 | 0.681 | 0.004 | T cells | 170.25 |
| *Itk* | 2.664926308 | 0.521 | 0.001 | T cells | 521 |
| *Bcl11b* | 2.547840868 | 0.465 | 0.001 | T cells | 465 |
| *Camk4* | 2.4509905 | 0.468 | 0.003 | T cells | 156 |
| *Lck* | 1.848040107 | 0.321 | 0.002 | T cells | 160.5 |
| *Gimap3* | 1.658678906 | 0.307 | 0.001 | T cells | 307 |

Pct.1 represents the proportion of cells expressing the marker gene within the current group of cells.

Pct.2 represents the proportion of cells expressing the marker gene in the remaining groups.
