# Supplementary material for: The suprachiasmatic nucleus regulates brown fat thermogenesis in male mice through an adrenergic receptor ADRB3-S100B signaling pathway
Source: PLoS Biol. 2025 Dec 4;23(12):e3003534. doi: 10.1371/journal.pbio.3003534 (PMC12688110; doi:10.1371/journal.pbio.3003534)
Supplement: S2 Table — (DOCX) [file pbio.3003534.s009.docx]

**S2 Table. The cell number and frequency for each cluster in snRNA-seq.**

| snRNA-seq | ZT16 SCN-lesioned | | ZT4 SCN-lesioned | | ZT16 Sham | | ZT4 Sham | |
| --- | --- | --- | --- | --- | --- | --- | --- | --- |
| cell type | cell num | freq | cell num | freq | cell num | freq | cell num | freq |
| T cells | 112 | 0.996885 | 88 | 0.70086 | 70 | 0.566756 | 91 | 0.893997 |
| B cells | 101 | 0.898976 | 60 | 0.477859 | 20 | 0.16193 | 112 | 1.100305 |
| Macrophages | 479 | 4.263462 | 736 | 5.861739 | 537 | 4.347826 | 321 | 3.153551 |
| Mural cells | 138 | 1.228304 | 185 | 1.473399 | 121 | 0.979678 | 238 | 2.338147 |
| Adipocytes | 8378 | 74.57054 | 9123 | 72.65849 | 9567 | 77.45932 | 7586 | 74.52598 |
| ASPC | 980 | 8.722741 | 1010 | 8.043963 | 946 | 7.659299 | 772 | 7.584242 |
| Neutrophils Monocytes DC | 178 | 1.584335 | 234 | 1.863651 | 236 | 1.910776 | 92 | 0.903822 |
| Endothelial cells | 863 | 7.681353 | 1076 | 8.569608 | 836 | 6.768683 | 942 | 9.254347 |
