# Supplementary material for: The suprachiasmatic nucleus regulates brown fat thermogenesis in male mice through an adrenergic receptor ADRB3-S100B signaling pathway
Source: PLoS Biol. 2025 Dec 4;23(12):e3003534. doi: 10.1371/journal.pbio.3003534 (PMC12688110; doi:10.1371/journal.pbio.3003534)
Supplement: S3 Table — (DOCX) [file pbio.3003534.s010.docx]

**S3 Table. IPA of differentially expressed genes..**

| ZT4TRF-sham vs SCN-lesioned | -log  (p-value) | Ratio | z-score | Molecules |
| --- | --- | --- | --- | --- |
| Circadian Rhythm Signaling | 6.51 | 0.0373 | NA | CIRBP,GNG11,HLF,NPR3,NR1D1,PER2,PER3,  PLCE1,PTGDS,RYR2 |
| Erythropoietin Signaling Pathway | 2.95 | 0.0284 | -2.236 | CCND1,HBA1,HBA2,HBB,Hbb-b1,Hbb-b2 |
| Iron homeostasis signaling pathway | 3.45 | 0.0365 | NA | HBA1,HBA2,HBB,Hbb-b1,Hbb-b2,SLC11A1 |
| S100 Family Signaling Pathway | 3.22 | 0.0143 | -2.111 | AHNAK,APLNR,CYBB,FCGR2A,GPRC5C,MMP12,  PLCE1,PTAFR,RYR2,S100B,Tpm4 |

| ZT16TRF-sham vs SCN-lesioned | -log  (p-value) | Ratio | z-score | Molecules |
| --- | --- | --- | --- | --- |
| Circadian Rhythm Signaling | 4.23 | 0.0933 | NA | ADCY2,ADCY9,ADRB1,BHLHE40,CACNA2D1,  CACNG1CACNG6,CRY1,DBP,GRINA,GSK3B,HLF,  LYN,NFIL3,NPR3,NR1D1,NR1D2,NR3C1,PER1,  PER2,PTGDS,RASD1,RORC,RYR2,SIRT1 |
| Erythropoietin Signaling Pathway | 2.39 | 0.0852 | -0.775 | ARNT,BCL2L1,CCND1,GSK3B,HBA1/HBA2,IRS2,  LTB,NFKBIA,PIK3C2B,PIK3R3,RAC2,RASD1,HBB,  Hbb-b1,Hbb-b2, |
| Iron homeostasis signaling pathway | 5.76 | 0.139 | NA | ALAS2,ARNT,ATP6V0D2,ATP6V0E1,FLVCR1,  GDF15,HBA1/HBA2,HIF3A,HJV,HMOX1,IL6R,  LRP1,SLC39A14,STEAP3,TF,TFRC,HBB,Hbb-b1,  Hbb-b2 |
| S100 Family Signaling Pathway | 3.18 | 0.0636 | -2.143 | ACKR3,ADGRE5,ADGRG1,ADGRG2,ADORA1,  ADRA1A,ADRB1,ADRB2,AHNAK,ANXA2,APLNR,ARNT,BTC,C3AR1,CACNA2D1,CACNG1,CACNG6,CASR,  CCR2,CCR5,CDKN1A,CX3CR1,CYBB,DLC1,FGFR2,  GSK3B,MC5R,MCAM,MMP12MMP15,MMP19,NR3C1,OXTR,PIK3C2B,PIK3R3,PTGER1,PTGER3,PTGIR,RAC2,  RYR2,S100A4,S100A6,S100B,SUCNR1,Tpm1,Tpm2,  Tpm4,TRAF3,WNT11 |
| p53 Signaling | 2.29 | 0.102 | 0.707 | BCL2L1,CCND1,CDKN1A,FAS,GADD45G,GSK3B,  PIK3C2B,PIK3R3,SIRT1,TP53INP1 |
| Senescence Pathway | 2.44 | 0.0736 | 1.414 | BHLHE40,CACNA2D1,CACNG1,CACNG6, SIRT1,  CAPN3,CAT,CCND1,CDK1,CDKN1A,ETS2,  GADD45G,HBP1,ING1,MAPKAPK3,PDK2,PDK4,  PIK3C2B,PIK3R3,PPP2R1B, PPP2R3A, RASD1 |
