# Supplementary material for: The suprachiasmatic nucleus regulates brown fat thermogenesis in male mice through an adrenergic receptor ADRB3-S100B signaling pathway
Source: PLoS Biol. 2025 Dec 4;23(12):e3003534. doi: 10.1371/journal.pbio.3003534 (PMC12688110; doi:10.1371/journal.pbio.3003534)
Supplement: S4 Table — (DOCX) [file pbio.3003534.s011.docx]

**S4 Table. Relative expression levels of *S100b* across each cluster under TRF-STE.**

| cell type**/**ZT4 | SCNx mean | Sham mean | p-value | pct.1 | pct.2 |
| --- | --- | --- | --- | --- | --- |
| Adipocytes | 0.105585 | 0.087720 | 1.13E-05 | 0.09 | 0.07 |
| ASPC | 0.069711 | 0.033167 | 0.006944 | 0.04 | 0.02 |
| B cells | 0.090071 | 0.031427 | 0.418765 | 0.05 | 0.03 |
| Endothelial cells | 0.069465 | 0.078829 | 0.325707 | 0.05 | 0.06 |
| Macrophages | 0.059586 | 0.038928 | 0.297917 | 0.05 | 0.03 |
| Mural cells | 0.105432 | 0.099854 | 0.934643 | 0.09 | 0.08 |
| Neutrophils Monocytes DC | 0.071663 | 0.036725 | 0.162459 | 0.06 | 0.02 |
| T cells | 0.146123 | 0.128257 | 0.904416 | 0.08 | 0.09 |
| ZT16 | SCNx mean | Sham mean | p-value | pct.1 | pct.2 |
| Adipocytes | 0.129674 | 0.091809 | 9.79E-16 | 0.11 | 0.08 |
| ASPC | 0.066495 | 0.050683 | 0.334114 | 0.04 | 0.03 |
| B cells | 0.031332 | 0.112926 | 0.628118 | 0.03 | 0.05 |
| Endothelial cells | 0.080247 | 0.081732 | 0.973595 | 0.06 | 0.06 |
| Macrophages | 0.079803 | 0.067478 | 0.640820 | 0.06 | 0.05 |
| Mural cells | 0.158396 | 0.061710 | 0.051434 | 0.12 | 0.05 |
| Neutrophils Monocytes DC | 0.058145 | 0.045639 | 0.872217 | 0.05 | 0.04 |
| T cells | 0.027590 | 0.089580 | 0.149531 | 0.03 | 0.07 |

Pct.1 represents the proportion of cells expressing the marker gene within the current group of cells.

Pct.2 represents the proportion of cells expressing the marker gene in the remaining groups.
