# Supplementary material for: The suprachiasmatic nucleus regulates brown fat thermogenesis in male mice through an adrenergic receptor ADRB3-S100B signaling pathway
Source: PLoS Biol. 2025 Dec 4;23(12):e3003534. doi: 10.1371/journal.pbio.3003534 (PMC12688110; doi:10.1371/journal.pbio.3003534)
Supplement: S5 Table — (DOCX) [file pbio.3003534.s012.docx]

**S5 Table. Quantitative RT-PCR primer information.**

| Gene | Primer | Sequences |  |
| --- | --- | --- | --- |
| *Adrb2* | Forward primer | ATGTCGGTTATCGTCCTGGC |  |
|  | Reverse primer | GGTTTGTAGTCGCTCGAACTTG |  |
| *Adrb3* | Forward primer | TCTCTGGCTTTGTGGTCGGA |  |
|  | Reverse primer | GTTGGTTATGGTCTGTAGTCTCG |  |
| *Ucp1* | Forward primer | GTGAACCCGACAACTTCCGAA |  |
|  | Reverse primer | TGCCAGGCAAGCTGAAACTC |  |
| *Pgc1α* | Forward primer | TATGGAGTGACATAGAGTGTGCT |  |
|  | Reverse primer | GTCGCTACACCACTTCAATCC |  |
| *S100b* | Forward primer | TGGTTGCCCTCATTGATGTCT |  |
|  | Reverse primer | CCCATCCCCATCTTCGTCC |  |
| *Cdk1α* | Forward primer | CCTGGTGATGTCCGACCTG |  |
|  | Reverse primer | CCATGAGCGCATCGCAATC |  |
| *Ccnd1* | Forward primer | GCGTACCCTGACACCAATCTC |  |
|  | Reverse primer | ACTTGAAGTAAGATACGGAGGGC |  |
| *Gadd45g* | Forward primer | GAAAGCACTGCACGAACTTCT |  |
|  | Reverse primer | CTTTGGCGGACTCGTAGACG |  |
| *Pkfl* | Forward primer | GGAGGCGAGAACATCAAGCC |  |
|  | Reverse primer | GCACTGCCAATAATGGTGCC |  |
| *Pkm* | Forward primer | CGCCTGGACATTGACTCTG |  |
|  | Reverse primer | GAAATTCAGCCGAGCCACATT |  |
| *Lctl* | Forward primer  Reverse primer | ACTACAAGTCAGCCACTACCG  CGCCGTATGCTACCTGGAG |  |
| *Actin* | Forward primer | ATGACCCAAGCCGAGAAGG |  |
|  | Reverse primer | CGGCCAAGTCTTAGAGTTGTTG |  |
| *Nd1* | Forward primer | CAGCCTGACCCATAGCCATA |  |
|  | Reverse primer | ATTCTCCTTCTGTCAGGTCGAA |  |
| *Cox1* | Forward primer | AGGCTTCACCCTAGATGACACA |  |
|  | Reverse primer | GTAGCGTCGTGGTATTCCTGAA |  |
| *Cyp2e1* | | Forward primer | CGTTGCCTTGCTTGTCTGGA |
|  |  | Reverse primer | AAGAAAGGAATTGGGAAAGGTCC |
| *Irf4* | | Forward primer | CCGACAGTGGTTGATCGACC |
|  |  | Reverse primer | CCTCACGATTGTAGTCCTGCTT |
| *Cyp4a10* | | Forward primer | TTCCCTGATGGACGCTCTTTA |
|  |  | Reverse primer | GCAAACCTGGAAGGGTCAAAC |
