# Supplementary material for: The suprachiasmatic nucleus regulates brown fat thermogenesis in male mice through an adrenergic receptor ADRB3-S100B signaling pathway
Source: PLoS Biol. 2025 Dec 4;23(12):e3003534. doi: 10.1371/journal.pbio.3003534 (PMC12688110; doi:10.1371/journal.pbio.3003534)
Supplement: S1 Raw Images — (PDF) [file pbio.3003534.s014.pdf]

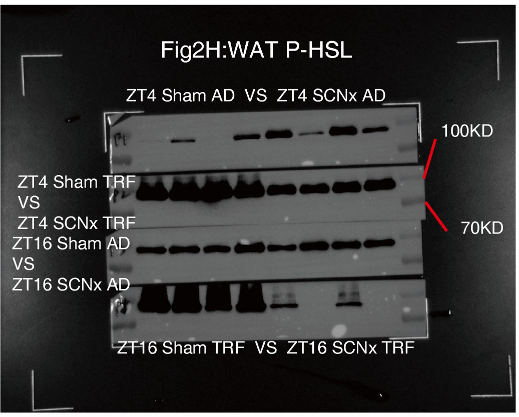

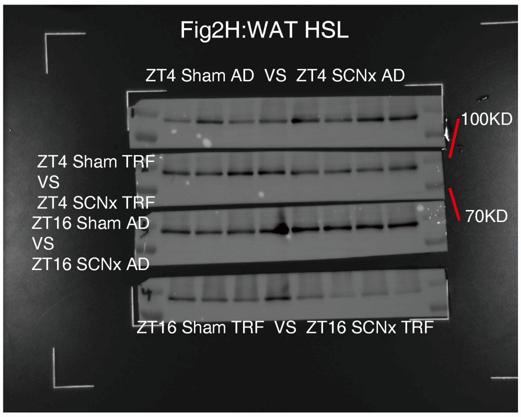

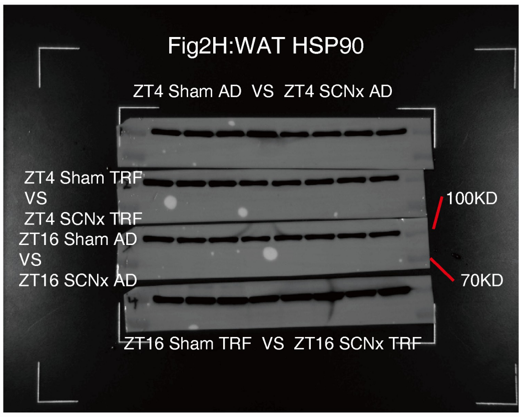

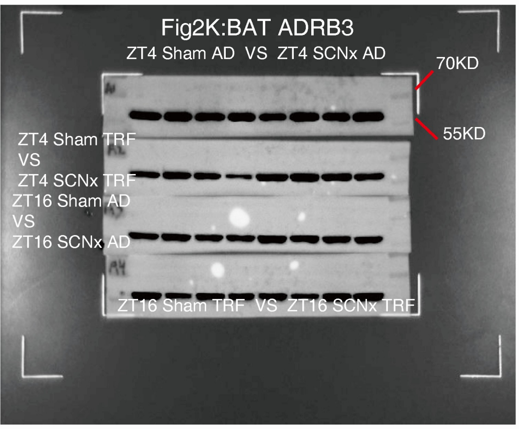

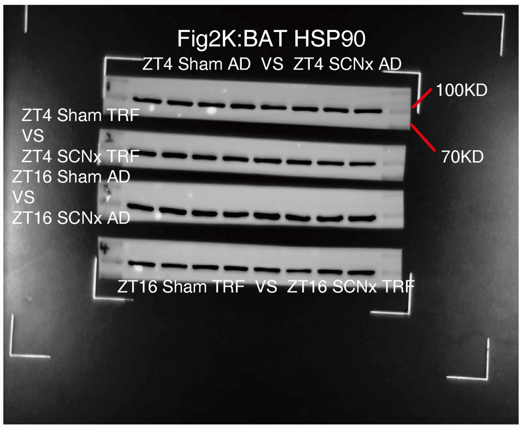

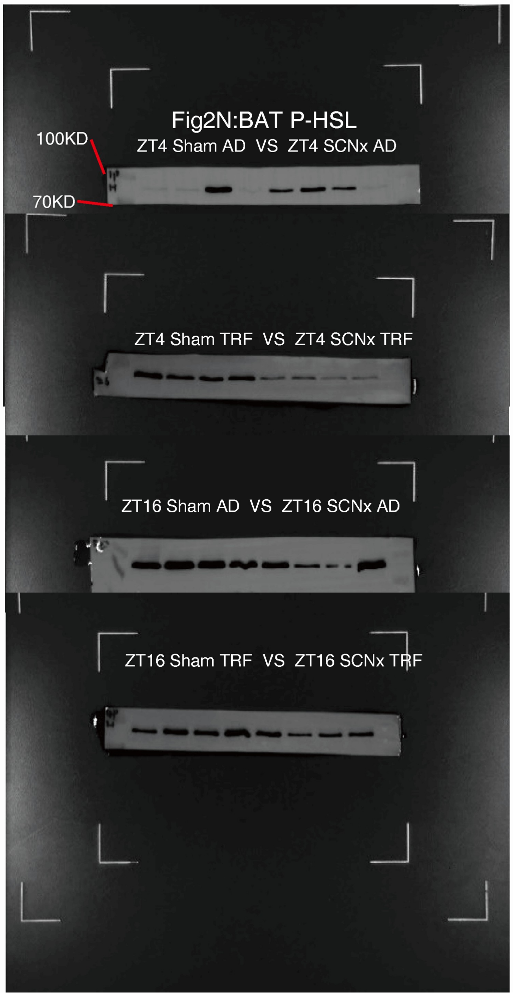

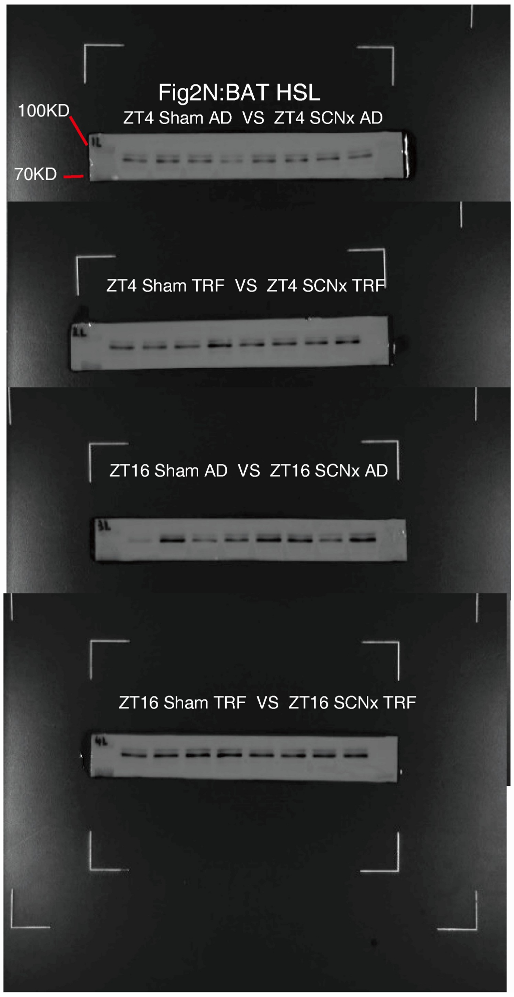

Fig2N:BAT HSP60

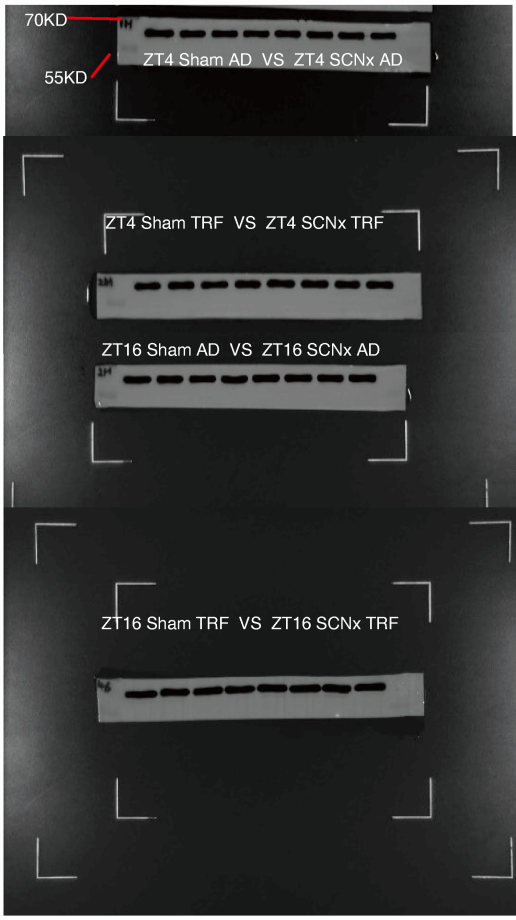

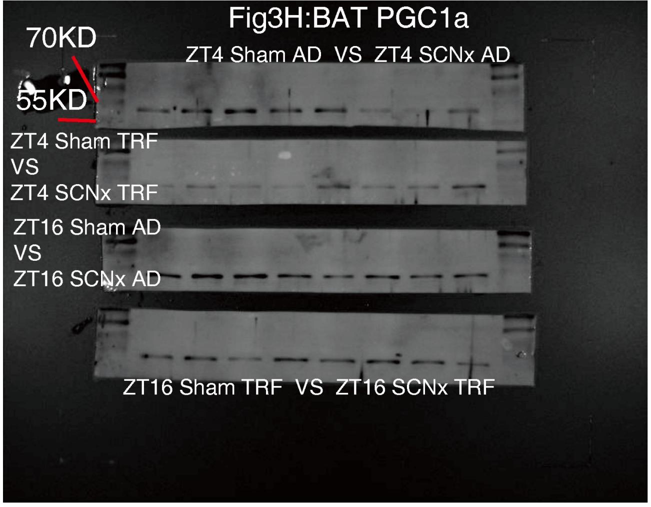
Fig3H:BAT PGC1a
70KD
55KD
ZT4 Sham AD VS ZT4 SCNx AD
ZT4 Sham TRF
VS
ZT4 SCNx TRF
ZT16 Sham AD
VS
ZT16 SCNx AD
ZT16 Sham TRF VS ZT16 SCNx TRF

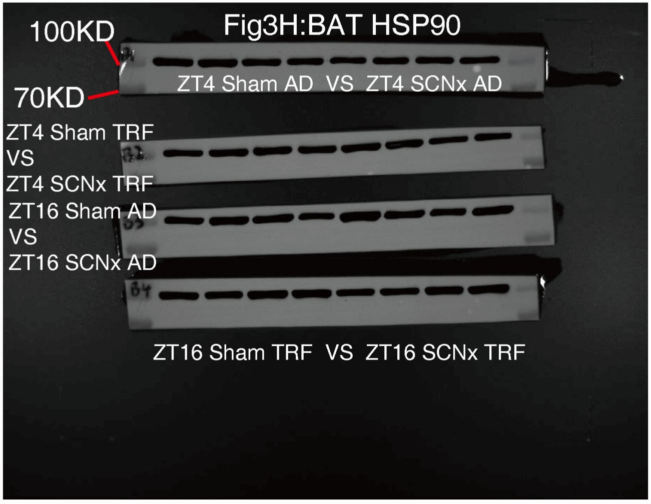
Fig3H:BAT HSP90
100KD
70KD
ZT4 Sham AD VS ZT4 SCNx AD
ZT4 Sham TRF
VS
ZT4 SCNx TRF
ZT16 Sham AD
VS
ZT16 SCNx AD
ZT16 Sham TRF VS ZT16 SCNx TRF

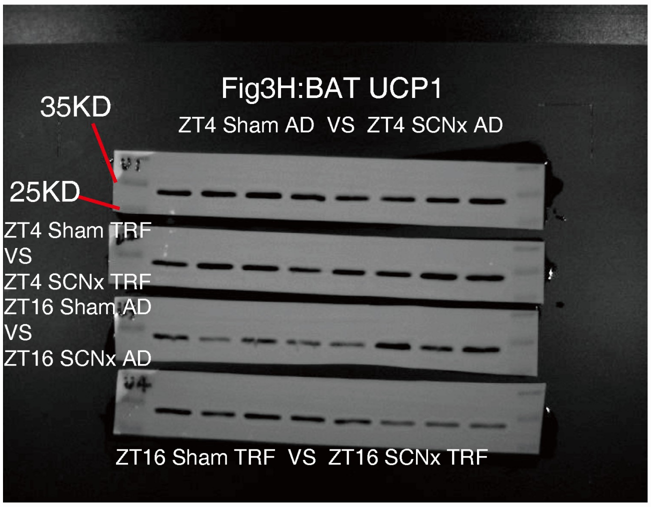
Fig3H:BAT UCP1
35KD
25KD
ZT4 Sham AD VS ZT4 SCNx AD
ZT4 Sham TRF
VS
ZT4 SCNx TRF
ZT16 Sham AD
VS
ZT16 SCNx AD
ZT16 Sham TRF VS ZT16 SCNx TRF

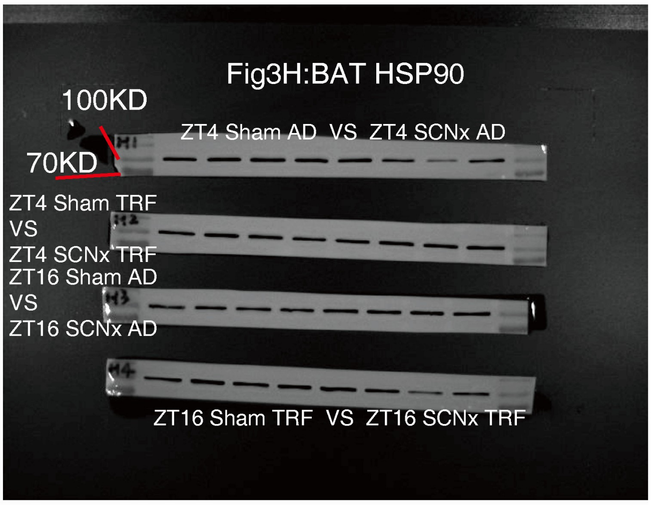
Fig3H:BAT HSP90
100KD
70KD
ZT4 Sham AD VS ZT4 SCNx AD
ZT4 Sham TRF
VS
ZT4 SCNx TRF
ZT16 Sham AD
VS
ZT16 SCNx AD
ZT16 Sham TRF VS ZT16 SCNx TRF

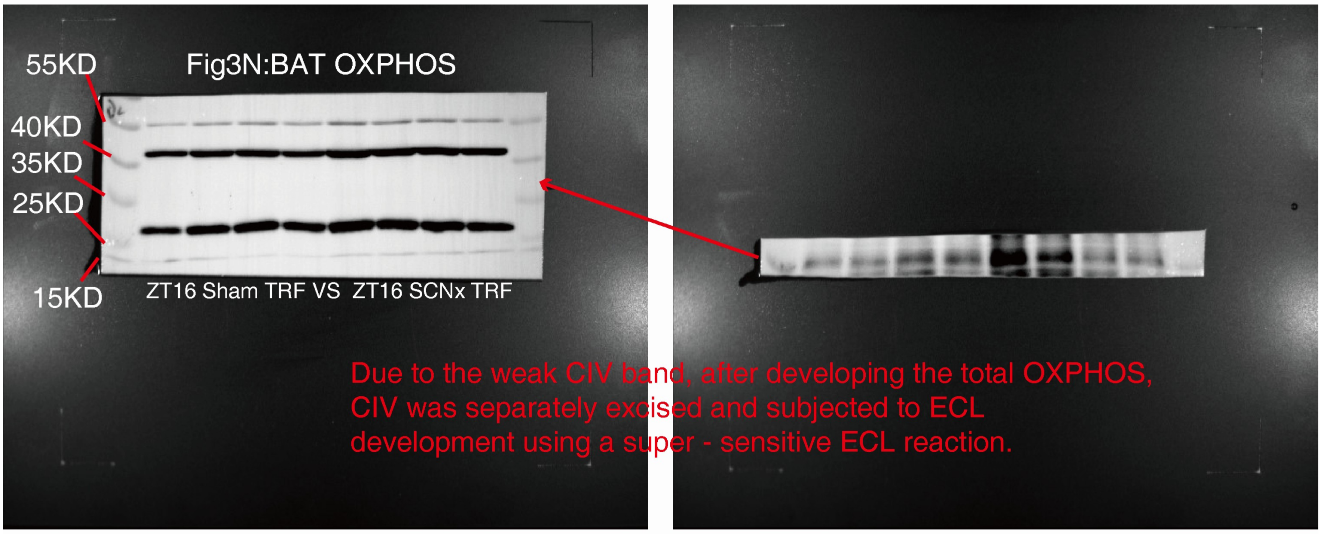
Fig3N:BAT OXPHOS
55KD
40KD
35KD
25KD
15KD
ZT16 Sham TRF VS ZT16 SCNx TRF
Due to the weak CIV band, after developing the total OXPHOS,
CIV was separately excised and subjected to ECL
development using a super - sensitive ECL reaction.

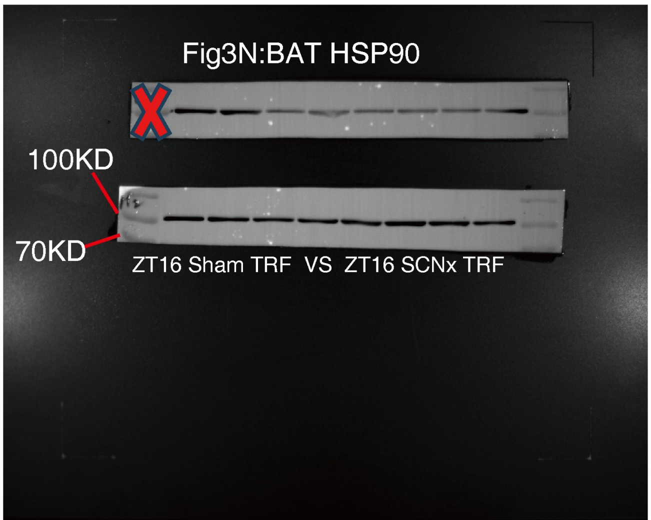
Fig3N:BAT HSP90
100KD
70KD
ZT16 Sham TRF VS ZT16 SCNx TRF

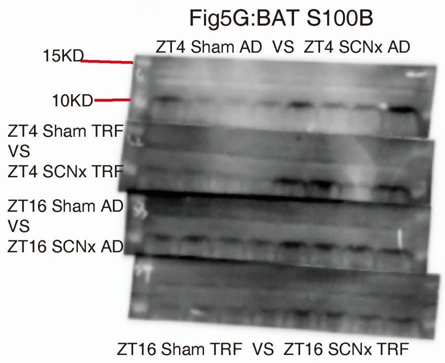
Fig5G:BAT S100B
ZT4 Sham AD VS ZT4 SCNx AD
15KD
10KD
ZT4 Sham TRF
VS
ZT4 SCNx TRF
ZT16 Sham AD
VS
ZT16 SCNx AD
ZT16 Sham TRF VS ZT16 SCNx TRF

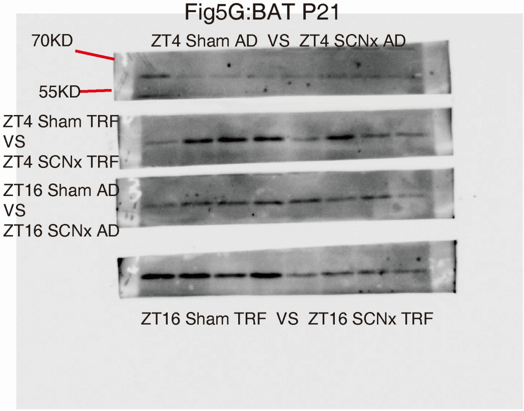
Fig5G:BAT P21
70KD
ZT4 Sham AD VS ZT4 SCNx AD
55KD
ZT4 Sham TRF
VS
ZT4 SCNx TRF
ZT16 Sham AD
VS
ZT16 SCNx AD
ZT16 Sham TRF VS ZT16 SCNx TRF

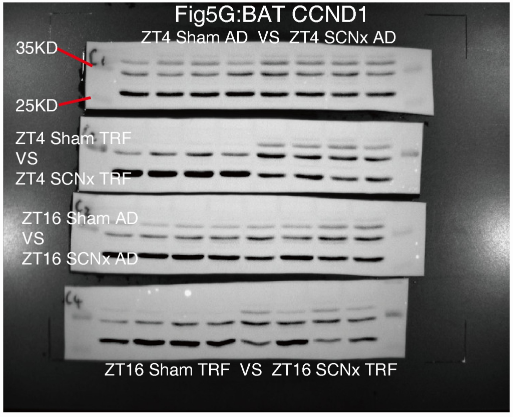
Fig5G:BAT CCND1
ZT4 Sham AD VS ZT4 SCNx AD
35KD
25KD
ZT4 Sham TRF
VS
ZT4 SCNx TRF
ZT16 Sham AD
VS
ZT16 SCNx AD
ZT16 Sham TRF VS ZT16 SCNx TRF

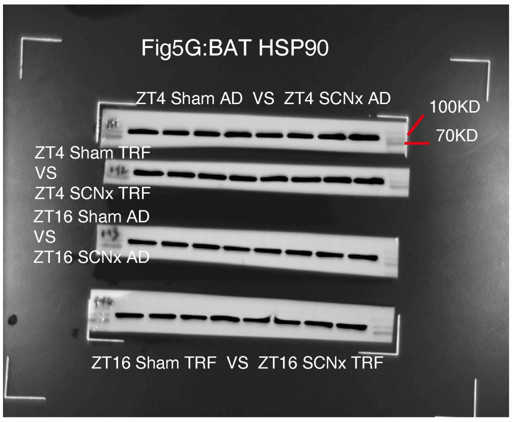
Fig5G:BAT HSP90
ZT4 Sham AD VS ZT4 SCNx AD
100KD
70KD
ZT4 Sham TRF
VS
ZT4 SCNx TRF
ZT16 Sham AD
VS
ZT16 SCNx AD
ZT16 Sham TRF VS ZT16 SCNx TRF

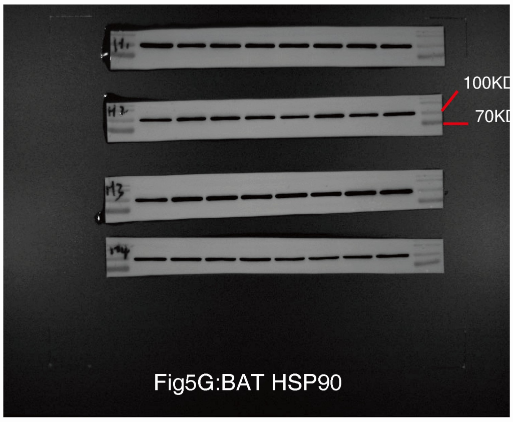
100KD
70KD
Fig5G:BAT HSP90

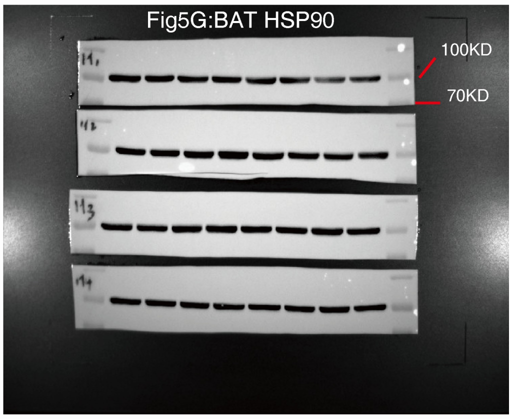
Fig5G:BAT HSP90
100KD
70KD

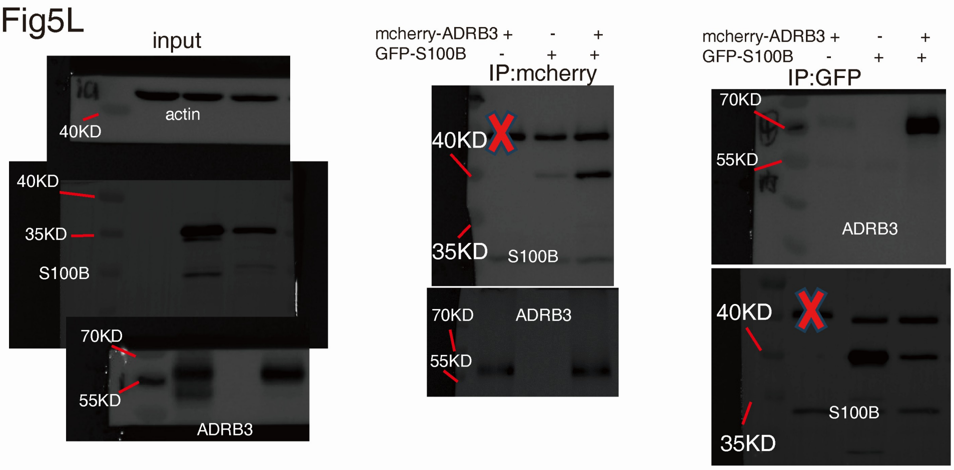
Fig5L
input
actin
40KD
40KD
35KD
S100B
70KD
55KD
ADRB3
mcherry-ADRB3 + - +
GFP-S100B - + +
IP:mcherry
40KD
35KD
S100B
70KD
ADRB3
55KD
mcherry-ADRB3 + - +
GFP-S100B - + +
IP:GFP
70KD
55KD
ADRB3
40KD
S100B
35KD

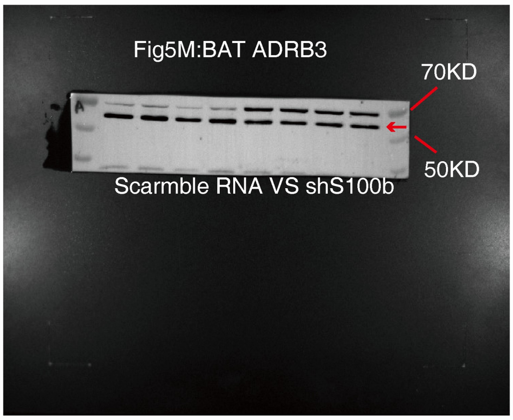
Fig5M:BAT ADRB3
70KD
50KD
Scarmble RNA VS shS100b

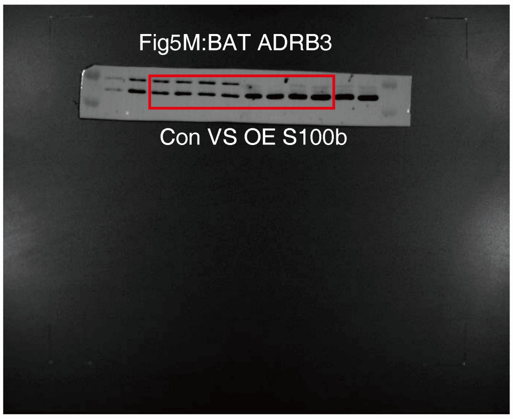
Fig5M:BAT ADRB3
Con VS OE S100b

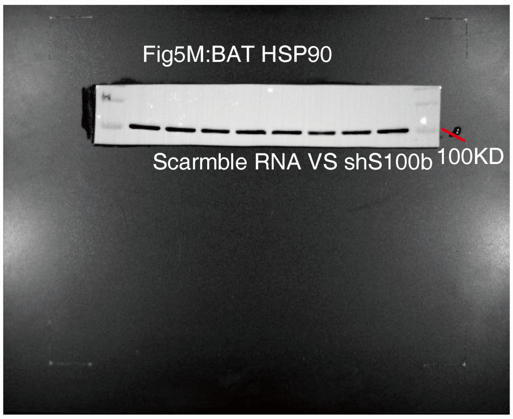
Fig5M:BAT HSP90
Scarmble RNA VS shS100b
100KD

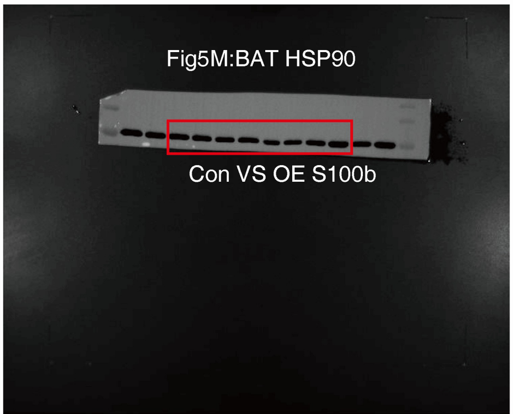
Fig5M:BAT HSP90
Con VS OE S100b

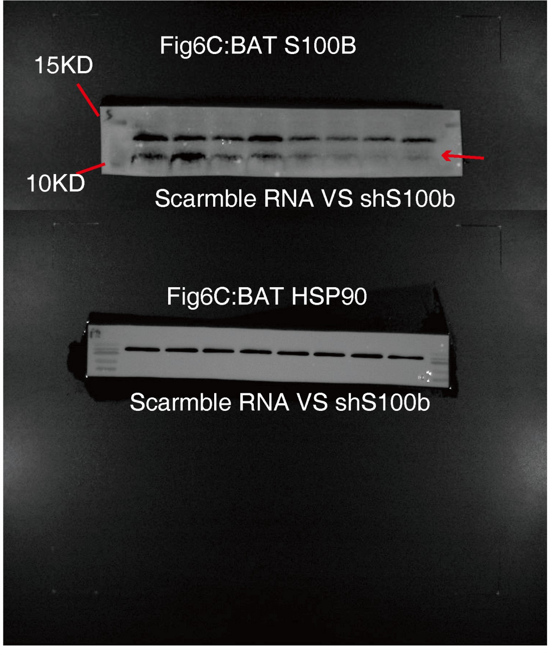
Fig6C:BAT S100B
15KD
10KD
Scarmble RNA VS shS100b
Fig6C:BAT HSP90
Scarmble RNA VS shS100b

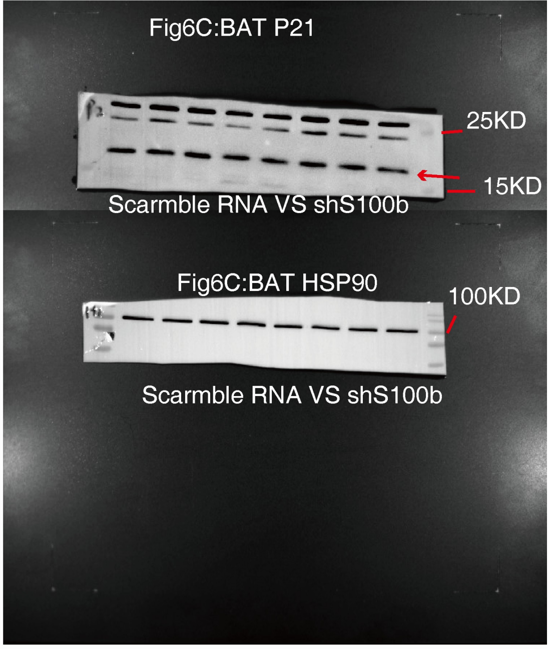
Fig6C:BAT P21
25KD
15KD
Scarmble RNA VS shS100b
Fig6C:BAT HSP90
100KD
Scarmble RNA VS shS100b

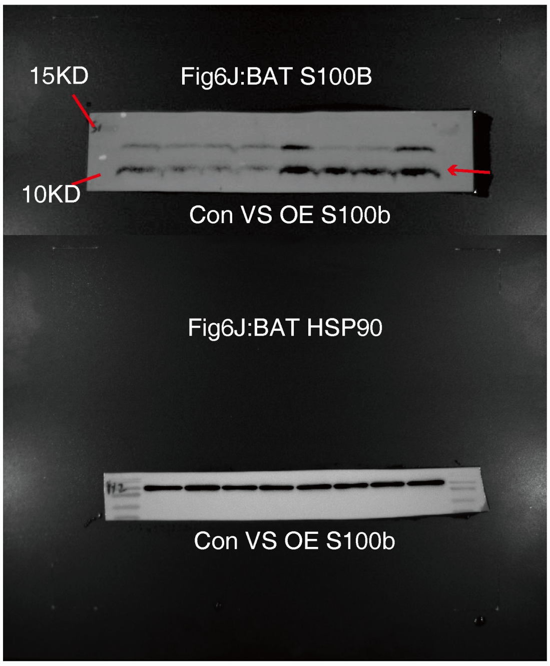
15KD
Fig6J:BAT S100B
10KD
Con VS OE S100b
Fig6J:BAT HSP90
Con VS OE S100b

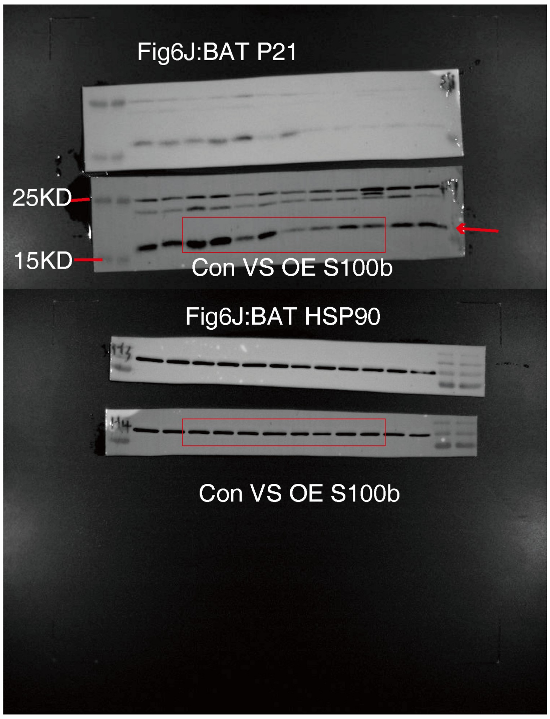
Fig6J:BAT P21
25KD
15KD
Con VS OE S100b
Fig6J:BAT HSP90
Con VS OE S100b

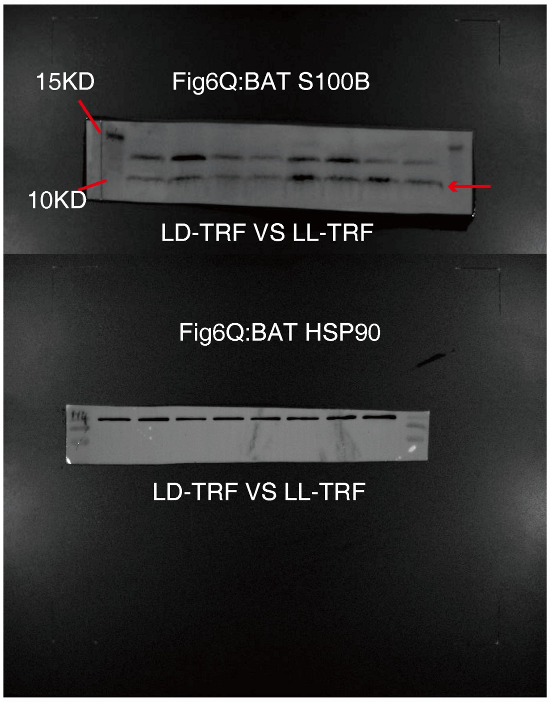
15KD
Fig6Q:BAT S100B
10KD
LD-TRF VS LL-TRF
Fig6Q:BAT HSP90
LD-TRF VS LL-TRF

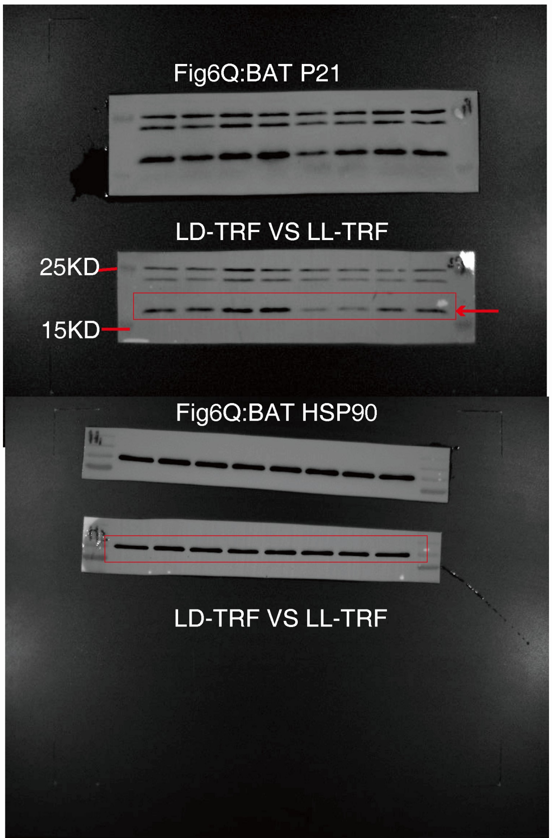
Fig6Q:BAT P21
LD-TRF VS LL-TRF
25KD
15KD
Fig6Q:BAT HSP90
LD-TRF VS LL-TRF

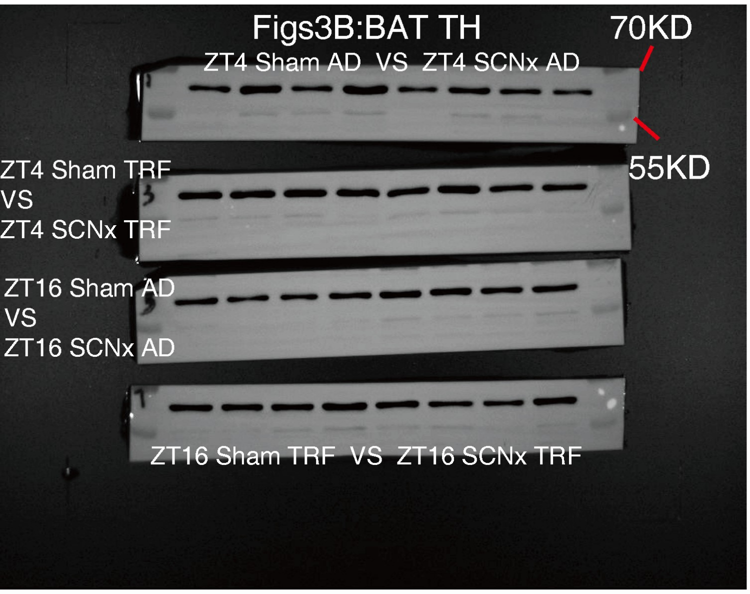
Figs3B:BAT TH
70KD
ZT4 Sham AD VS ZT4 SCNx AD
55KD
ZT4 Sham TRF
VS
ZT4 SCNx TRF
ZT16 Sham AD
VS
ZT16 SCNx AD
ZT16 Sham TRF VS ZT16 SCNx TRF

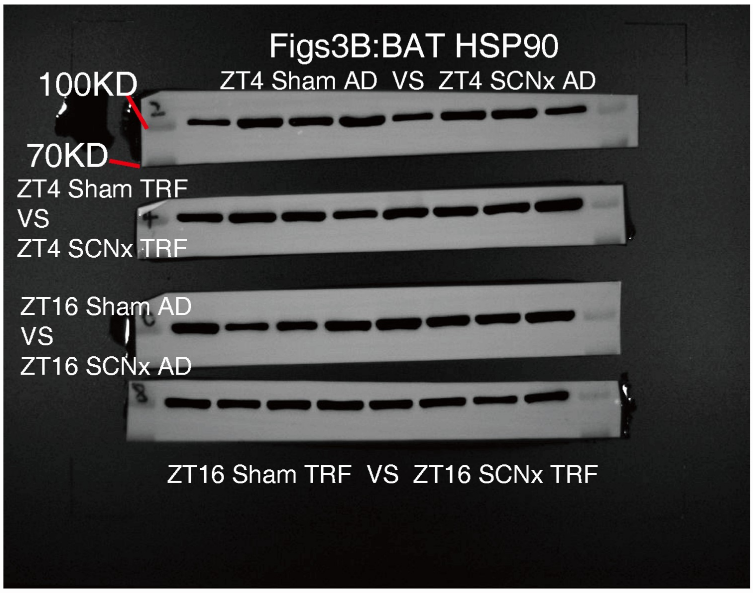
Figs3B:BAT HSP90
100KD
ZT4 Sham AD VS ZT4 SCNx AD
70KD
ZT4 Sham TRF
VS
ZT4 SCNx TRF
ZT16 Sham AD
VS
ZT16 SCNx AD
ZT16 Sham TRF VS ZT16 SCNx TRF

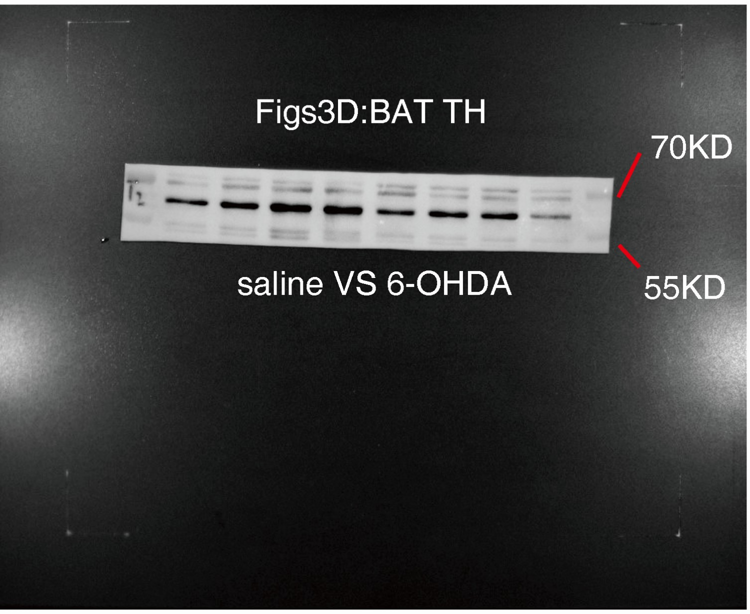
Figs3D:BAT TH
70KD
saline VS 6-OHDA
55KD

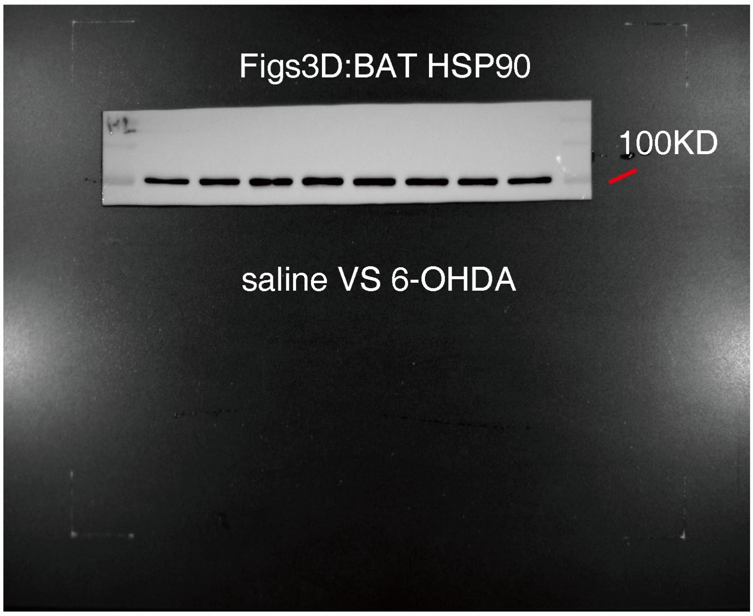
Figs3D:BAT HSP90
100KD
saline VS 6-OHDA

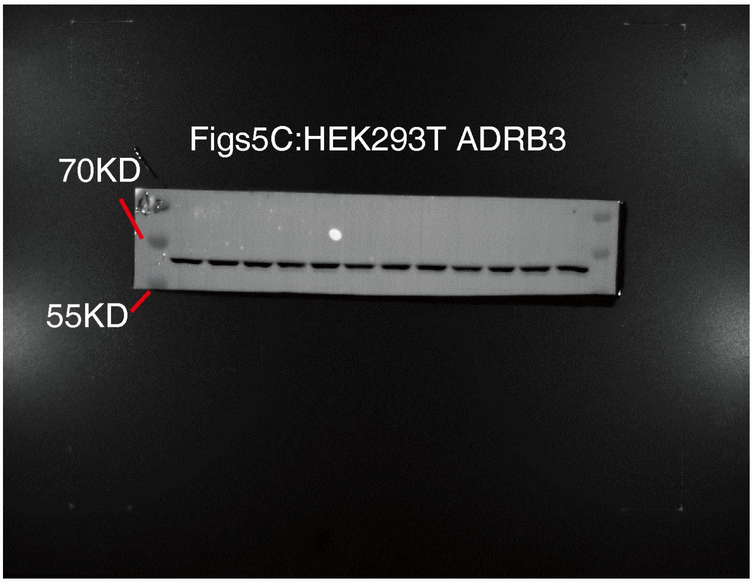
Figs5C:HEK293T ADRB3
70KD
55KD

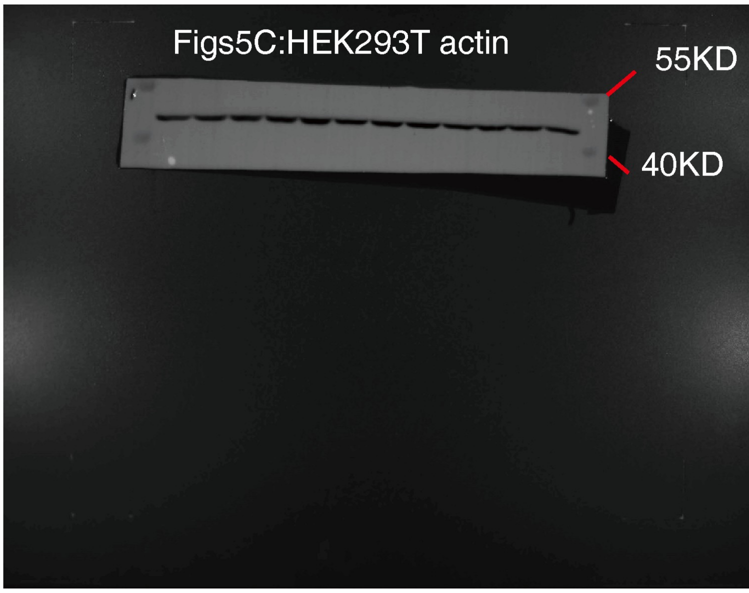
Figs5C:HEK293T actin
55KD
40KD
