# Supplementary material for: The suprachiasmatic nucleus regulates brown fat thermogenesis in male mice through an adrenergic receptor ADRB3-S100B signaling pathway
Source: PLoS Biol. 2025 Dec 4;23(12):e3003534. doi: 10.1371/journal.pbio.3003534 (PMC12688110; doi:10.1371/journal.pbio.3003534)
Supplement: Attachment — Submitted filename: Response to editor and reveiwers.docx [file pbio.3003534.s015.docx]

Dear Luke,

Thank you for your detailed editorial summary and for the opportunity to revise our manuscript. We appreciate the insightful comments from you, the Academic Editor, and all three reviewers. We have now carefully revised our manuscript to address each point raised and believe the revised version substantially improves the clarity, rigor, and interpretability of the study.

As requested, we have provided a **point-by-point response** to all reviewer comments in the accompanying document. In parallel, we have updated the manuscript with **track changes** and highlighted below the most significant additions and revisions:

**Summary of Major Revisions and Additions**

**New Control Data (Reviewer 1):**

We now include core body temperature data under standard housing conditions (ad libitum feeding vs. TRF), initially published in our previous work. These have been re-analyzed and aligned with the current dataset in the revised Fig 1A, clearly demonstrating the physiological impact of TRF in intact mice.

**Clarification of the SCN-S100B Mechanism (Reviewer 1):**

We have expanded the Discussion to clarify the role of S100B as a nutrient-sensitive amplifier of sympathetic signaling, distinct from canonical cold sensors. This resolves the apparent discrepancy between S100B's nutrient sensitivity and its thermogenic function under SCN regulation.

**Clarification of Multiple SCN Disruption Models (Reviewer 1):**

To exclude confounding artefacts from lesioning, we used three independent SCN disruption models (LL, NMDA and caspase-3) and showed convergent elevation of S100B and preserved thermogenesis. These data support the robustness and reproducibility of our findings. To enhance this result, we present Fig 6 O-V as an independent Fig 7.

**Additional Experiments (Reviewer 2):**

Added quantification of lipid droplet size in eWAT and Oil Red O staining in BAT (S2A and S2D Fig).

Included OCR measurements of mitochondria isolated from 6-OHDA-treated in sham and SCN lesioned mice (S3M and S3N Fig).

Clarified snRNA-seq findings, emphasizing that S100B expression was elevated only in brown adipocytes, not in other cell types (S5A and S5B Fig, S4 Table).

Provided PDGFRα immunostaining to validate EdU⁺ cells as preadipocytes (S4G and S4H Fig).

**Clarification of Analysis (Reviewer 3):**

Corrected the description of our General Linear Model to specify that body weight was used as a covariate (Fig 1K, S1H Fig, Page 5).

**Added a narrative explanation of Figure 7H and the Figure Legend (Reviewers 2 and 3).**

We updated Figure 7H and its legend to include TRF timing (ZT16-ZT20) and environmental context. The revised legend now reads:

*“(H) Working model illustrating the SCN-ADRB3-S100B axis in BAT during TRF conducted at ZT16-ZT20 in a subthermoneutral environment, the SCN regulates BAT thermogenic plasticity via SNS output. SCN lesioning enhances ADRB3 signaling and S100B expression, which together form a positive feedback loop that amplifies β3-adrenergic sensitivity, promotes preadipocyte proliferation, suppresses senescence, and sustains glucose-driven thermogenesis. Created with BioRender.com*.”

**Sex of Experimental Animals:**

All experiments were performed in male mice to avoid estrous cycle-associated variability. This has been clearly stated in Methods, and we have updated the title accordingly:

*Suprachiasmatic nucleus regulation of brown fat thermogenesis via the ADRB3-S100B axis in male mice*

We are grateful for the opportunity to strengthen the manuscript and address the constructive critiques provided. We hope that the revised version meets the expectations of the editorial team and reviewers. Please let us know if further clarification or additional experiments are required, and we remain fully committed to ensuring the highest quality for this work.

Sincerely,

Ying

On behalf of all authors

**Point-to-point response**

*Reviewer #1: Zheng et al present a study whereby mice were kept at sub thermal conditions, changed into a new cage without food at ZT 20 (four hours before light on), kept without food until the next ZT 16, then given food till ZT 20. Intact (Sham lesioned) animals exposed to these conditions were compared with those having a Suprachiasmatic Nucleus (SCN) lesion. No comparison was made between intact animals in normal conditions and the food-restricted groups to show the impact of this food restriction on the normal physiology.*

**Response:**
We thank the reviewer for this important point. Our current work builds on our previous findings (Zhang et al., *Science Bulletin*, 2020, Fig 3), where we first demonstrated that intact mice exhibit significant hypothermia under combined thermal and nutritional stress, while SCN lesioning mitigates this phenotype.

To strengthen the present study, we have now included data showing core body temperature changes in intact mice under conventional housing conditions when switched from *ad libitum* feeding to time-restricted feeding (TRF). These results are now highlighted in the revised Fig 1A, to clearly illustrate the physiological impact of TRF on normal intact animals.

We have also added text in the revised clean version: *“Under conventional housing conditions (CC, 23-25°C), WT mice exhibited a modest CBT reduction when switched from ad libitum to TRF (Fig 1A). And at 21 °C, sham-operated mice (sham mice) displayed a much larger drop in CBT and BAT temperature upon food withdrawal at ZT20, consistent with a hypothermic adaptation to fasting (Fig 1A and 1B). By contrast, SCN-lesioned (SCNx) mice (S1A Fig, described in methods) showed attenuated hypothermia during the second TRF cycle, maintaining significantly sustained CBT and BAT temperatures than sham mice at 21°C or WT under CC (Fig 1A and 1B).”* on page 5 lines 10 to 17.

*Next, the authors demonstrate that the SCN influences brown adipose tissue (BAT) through the sympathetic nervous system. Followed by an analysis of the involved genes in BAT using SCN-intact and lesioned animals. Hereafter, the study focuses on the S100B calcium-binding protein.*

*A 48-hour fast completely suppressed S100b, whereas high-fat diet (HFD) feeding significantly increased its expression. Interestingly, thermoneutral acclimation (30 °C) markedly suppressed S100b, while cold exposure (4 °C) did not influence S100b. These findings demonstrate that S100b is especially regulated by nutrient availability rather than cold stress. This seems in contrast with the conclusion of the paper that SCN influences BAT thermogenesis via S100B signaling.*

**Response:**
We thank the reviewer for this insightful observation. Indeed, our data clearly demonstrated that *S100b* expression is more strongly modulated by nutrient availability than by cold exposure, which we highlight as a ***key novel finding*** of our study (Fig 5A and 5B). While classical thermogenic genes like *Ucp1* are directly induced by cold, *S100b* expression remained unchanged between 21°C and 4°C. In contrast, *S100b* was significantly downregulated by fasting and upregulated by HFD, even at both 21°C and thermoneutrality (30°C), indicating that *S100b* is tightly related to metabolic fuel status rather than ambient temperature.

This nutrient sensitivity, however, does not contradict the conclusion that SCN regulates thermogenesis via S100B, but rather redefines the functional context in which S100B operates. Our findings indicate that S100B is not a primary cold sensor, but instead serves as a nutrient-sensitive amplifier of β3-adrenergic signaling, which is under SCN control.

Several lines of evidence support this interpretation:

1. **Functional Synergy**: In vitro, recombinant S100B enhances glycolytic activity (ECAR) and potentiates thermogenic responses when combined with β3-adrenergic stimulation (CL-316243) (Fig 5O-5P), indicating its cooperative role with sympathetic signaling.
2. **In Vivo Causality:** *S100b* knockdown in SCN-lesioned mice abolishes their thermogenic resilience (Fig 6G), while *S100b* overexpression in wild-type mice restores resistance to TRF-induced hypothermia (Fig 6N), demonstrating necessity and sufficiency.
3. **SCN and SNS Dependence:** *S100b* expression is suppressed by SCN (Figs 4A, 5F, S5A and S5B), sympathetic denervation (6-OHDA; Fig 3E), and β3-adrenergic blockade (SR59230A) abolishes both *S100b* expression differences (Fig 5I) and the associated thermogenic phenotype (Fig 3F), firmly linking *S100b* regulation to SCN-mediated sympathetic output.

To clarify this conceptual integration in the revised manuscript, we have revised the discussion (Page 18, lines 2 to 16 in the revised clean version) as follows:

“*A significant contribution of this study is the identification of the ADRB3-S100B axis as a key effector of SCN-mediated BAT regulation. S100B expression is determined by nutrient availability under SCN control, being suppressed by fasting and thermoneutrality, and upregulated by HFD feeding, its functional impact on thermogenesis only becomes evident in the context of activation of β3-adrenergic signaling. We show that S100B physically interacts with ADRB3, increases receptor abundance, and enhances glycolytic activity in brown adipocytes, especially when combined with β3-adrenergic agonists. Although the mechanism by which S100B affects ADRB3 phosphorylation remains to be elucidated (35, 36, 41), their synergistic impact on ECAR suggests a sensitizing feedback loop that amplifies β3-adrenergic signaling. Unlike canonical thermogenic genes such as Ucp1, which respond to cold, S100b is nutrient-sensitive, downregulated by fasting and thermoneutrality, yet upregulated by HFD, highlighting its role as a metabolic amplifier attuned to energy supply. Thus, S100B acts not as a direct cold sensor but as a nutrient-sensitive amplifier of sympathetic input*”.

We hope this revision addresses the reviewer’s concern and clarifies the novel positioning of S100B as a metabolic/nutritional rather than thermal signal integrator under SCN regulation.

*The reason for discrepancy might be that the authors only use SCN lesioning or by disrupting SCN functioning in other ways. The observation that SCN lesioning promotes S100B activation might be just related with the fact that SCN lesioning is known to prevent the sleep associated temperature decrease.*

*Critique: For this reviewer it was very difficult to understand the rationale of the present study. Since the study of Liu et al (ref 12) it is known that lesioning the SCN results in the prevention of the sleep associated temperature dip, SCN lesioning keeps the temperature high. This means that the SCN does not stimulate so much the sympathetic output to BAT but rather inhibits it, resulting in a dip in body temperature in the beginning of the sleep phase. Consequently, also the fasting induced temperature dip in the beginning of the sleep phase does not occur after SCN lesioning.*

**Response:**
We thank the reviewer for this important comment and the opportunity to clarify the rationale behind our study. We agree that SCN lesioning is known to prevent the normal circadian drop in core body temperature during the sleep phase, as established by Liu et al (Ref 12). However, our findings extend beyond this well-described phenomenon and instead focus on *a distinct and pathophysiological form of hypothermia* observed under time-restricted feeding in a subthermoneutral environment (TRF-STE), which goes well beyond the scope of the normal sleep-associated temperature dip.

Specifically, the circadian temperature dip described in previous work is modest (~1.5-2 °C) and remains within the physiological range. In contrast, mice under TRF-STE conditions exhibit a dramatic and progressive drop in core body temperature, reaching ~34 °C by day 1 and as low as 22 °C by days 3-4, levels that approach the threshold of torpor and often result in death if sustained (Zhang et al., 2020). This extreme hypothermic response is not merely a byproduct of sleep onset but instead represents a maladaptive energetic response leading to a homeostatic crisis under combined nutritional and thermal stress.

To clarify this distinction, we reanalyzed body temperature data from mice undergoing TRF initiated at different zeitgeber times (ZT4-8, ZT10-14 and ZT22-2). Regardless of the circadian phase at which feeding was initiated, hypothermia consistently coincided with periods of food unavailability, not with sleep onset (revised Fig S1B). We now highlight this analysis in the revised manuscript (in revise page 5, lines 17-22) to emphasize that SCN lesioning prevents stress-induced pathological hypothermia, rather than merely blunting a physiological circadian fluctuation.

This experimental paradigm distinction is central to the rationale of our study. Under TRF-STE, intact SCN signaling paradoxically suppresses thermogenesis, contributing to energy collapse. In contrast, removal of SCN function removes the adaptive thermogenic brake and allows BAT to sustain thermogenic activity, maintain body temperature, and avoid lethality. These findings uncover a previously unrecognized role of the SCN in constraining thermogenic flexibility, aiming to conserve energy under metabolic stress and help redefine how circadian regulation interacts with adaptive sympathetic and nutrient-responsive pathways in brown adipose tissue.

*Furthermore, it has already been known for long that the SCN can influence BAT physiology by influencing the sympathetic innervation of BAT(Bamshad et al A.J.Phys. 1999).*

**Response:**
We thank the reviewer for pointing this out. Indeed, we fully acknowledge the foundational work by Bamshad et al. (Am. J. Physiol., 1999) which is cited in our manuscript. They used pseudorabies virus (PRV) retrograde tracing to reveal the multisynaptic sympathetic circuitry projecting to brown adipose tissue (BAT), including key nodes in the hypothalamus such as the SCN, PVN and DMH. As the reviewer notes, this study clearly established that the SCN is anatomically connected to BAT via the sympathetic nervous system.

Our study builds directly upon these anatomical insights by extending them into a functional context. Specifically, we confirm SCN-BAT connectivity via anterograde viral tracing (Fig 3A) and, critically, we provide physiological and molecular evidence that SCN signaling actively regulates BAT thermogenesis under metabolic stress.

In contrast to earlier anatomical work, our data show that SCN lesioning leads to a dissociation between whole-body energy expenditure and BAT thermogenic output, which is preserved in the face of energetic stress. We identify a mechanistic pathway, the ADRB3-S100B signaling axis that mediates this functional regulation. This axis is responsive to sympathetic tone and nutrient cues and acts downstream of SCN activity to promote adaptive thermogenesis and preadipocyte proliferation.

Thus, while the anatomical influence of the SCN on BAT has been known, our findings contribute a novel mechanistic understanding of how SCN activity functionally modulates BAT physiology under real-world stressors, such as food scarcity and cold exposure. We clarified this conceptual advance in the Discussion section (Page 18, line 40-page 19, line 2)

*Furthermore, the imposed acute(!) change in food availability, as used in the present paper, influences many SCN associated systems that normally regulate the body temperature depending on the availability of food (see e.g. a review of Mendez-Hernandez et al 2020 Obesity).*

*Suggestion: The authors indicate several possibilities to promote an interest in protein S100B in association with the functioning of the SCN. I would suggest that the authors use a more straightforward model in which the known functions of the SCN: deepening the temperature trough during fasting at ZT 0-2 is associated with an increase in temperature to normal high levels during activity. This approach would have the possibility to reveal the functioning of S100B in association with the functioning of the SCN. Now the authors use a model (SCN-lesioning) that was very useful in 2002 (ref12) but since then it was shown that the SCN influences sympathetically BAT (Bamshad), manages in interaction with the arcuate nucleus the daily temperature increase and decrease (Guzman-Ruiz J. Neurosci. 2015). When the authors want to clarify a role for S100B in the temperature regulation of the SCN and brown adipose tissue metabolism, it would be more logical to focus on the normal daily*

*temperature changes associated with the SCN driven anticipation of activity and rest that are more pronounced during fasting conditions (REF 12).*

**Response:**
We understand the reviewer’s concern regarding the specificity of S100B regulation. To avoid an artefact of SCN lesion, we used multiple complementary models of SCN dysfunction in our manuscript:

1. SCN lesioning,
2. Constant light exposure (LL),
3. NMDA-induced excitotoxicity, and
4. Caspase-3-mediated GABAergic neuron ablation.

In all cases, BAT showed elevated S100B expression, increased proliferation (*Ccnd1*), and suppressed senescence (*Cdkn1a*), paralleling the thermoprotective phenotype. These findings argue against S100B induction being a nonspecific artefact of lesioning or acute feeding stress. Instead, they support a robust link between SCN disruption and nutrition-induced S100B-driven BAT remodeling. To enhance this result, we present Fig 6 O-V as an independent Fig 7.

Given the consistent changes of S100B across different mouse models, we also overexpressed S100B in mice with an intact SCN. We observed significant differences in body temperature under TRF-STE, further demonstrating the link between elevated BAT S100B and thermogenesis.

Furthermore, 6-OHDA denervation and ADRB3 blockade abolished both S100B upregulation and thermogenic protection in SCN-lesioned mice, confirming that intact sympathetic input is required for S100B-mediated effects.

Finally, our structural and biochemical studies demonstrated a direct interaction between S100B and ADRB3, with S100B modulating receptor abundance and sensitivity. This functional coupling further supports S100B as an effector of SCN-dependent sympathetic regulation, not a byproduct of lesioning.

We have clarified these points in the revised Discussion to highlight that S100B elevation is a reproducible and mechanistically relevant outcome of SCN dysfunction across multiple models.

We have added the physiological relevance of the study, rationales for the approaches, and limitations in the revised discussion on Page 18, lines 2-19, lines 20-37.

*Reviewer #2: In this study, the authors investigate the role of the suprachiasmatic nucleus (SCN) in the regulation of brown adipose tissue (BAT) thermogenesis under conditions of time-restricted feeding in subthermoneutral environments (TRF-STE). They show that SCN lesioning preserves BAT thermogenic capacity while disrupting substrate using, specifically by impairing lipid mobilization and promoting glucose-driven thermogenesis. These effects are mediated through alterations in sympathetic nervous system signaling, indicating that the SCN modulates thermogenesis via this way. They also demonstrate that SCN lesioning reverses TRF-STE-induced cellular senescence in BAT and promotes cell proliferation through the S100B, a protein identified as a nutrient-sensitive effector. S100B amplifies β3-adrenergic signaling and enhances thermogenic capacity. The study further demonstrates that S100B is both necessary and sufficient to mediate SCN-dependent thermogenic resilience in BAT,*

*highlighting its central role in the metabolic adaptation of this tissue. The authors show that S100B physically interacts with the β3-adrenergic receptor (ADRB3), modulating its abundance and potentially enhancing its signaling responsiveness. Overall, their results identify the ADRB3-S100B axis as a key adaptive mechanism in BAT following SCN disruption.*

*The work is of high quality, as is the writing of the manuscript and the presentation of the data. The amount of work is impressive, with a wide variety of models and methods to support the conclusions. Here are some minor comments that are nonetheless worth addressing:*

*In general, for most experiments, the authors should indicate in the text or figure legend how long after SCN ablation the experiments were performed. For example, changes in body composition take time and the data in Figure 1 G-I should be supplemented with the time taken to obtain such changes after SCN ablation. How do the authors explain that actograms show complete desynchronization of the circadian clock, while EE and temperature still show a circadian profile in SCNx animals? Does food intake also retain a circadian profile?*

**Response:**

We appreciate this important point. All experimental mice were 8 weeks old at the start. Following SCN lesioning, mice recovered for 1 week, then underwent wheel-running screening for 1 week. Mice showing complete loss of circadian rhythmicity without abrupt body weight gain were selected and housed in a temperature-controlled 21°C chamber for 2 weeks of acclimation before TRF experiments. We have now specified it in the figure legend (Fig 1).

As the reviewer notes, the persistence of rhythmicity in EE and body temperature reflects food-entrained rhythms under TRF, consistent with prior studies showing that feeding can impose rhythmicity on metabolic outputs even in SCN-lesioned mice. Under wheel-running screening with ad libitum food, no such rhythms were detectable.

*- Fig 2: The increase in lipid droplet size in SCNx mice is not evident, particularly in Ad-STE. The authors should perform quantification to support their conclusion of larger lipid droplets in SCN-injured MTDs in both Ad-STE and TRF- STE. Oil-Red-O staining would also be informative.*

**Response:** Thank you for this suggestion. We have now quantified adipocyte area distribution in eWAT and included these results in S2A Fig. Although our attempts at Oil-Red-O staining of eWAT were unsuccessful, Oil-Red-O staining was successful in BAT, and we quantified lipid droplet area in BAT as shown in S2D Fig. These new data strengthen our conclusion that SCN lesioning promotes lipid storage in BAT.

*Fig 3: the authors should indicate the number of samples used in this experiment and quantify the signals in each animal. To conclude that SCN modulates thermogenic capacity via sympathetic signaling, OCR should be measured in mitochondria isolated from the interscapular BAT of sham-6-OHDA and SCNx-6-OHDA mice, and in sham and SCNx mice treated with the ADRB3 antagonist.*

**Response:**

We appreciate the reviewer’s thoughtful comments and agree that quantification and appropriate functional validation are essential.

For the 3D sympathetic innervation analysis, we acknowledge that our sample size was limited. Whole-body BAT clearing and sympathetic innervation reconstruction are highly resource-intensive procedures (~$3,400 per specimen), which constrained throughput. In a preliminary cohort (n = 12), we observed a <10% coefficient of variation in interscapular BAT mass within groups. Based on this consistency and following the manufacturer's guidance, we processed one fully cleared sample per group as an initial qualitative screen. The observed differences in sympathetic innervation were robust, and thus, additional replication was not pursued at this stage. We now explicitly state this limitation (n = 1 per group) in the revised figure caption.

To address the reviewer’s request for functional validation, we performed mitochondrial respiration analysis (OCR) using Seahorse metabolic flux assays in mitochondria isolated from interscapular BAT. These new data are now included in the revised S3M and S3N Fig. The results show that the elevated mitochondrial respiration in SCNx mice is completely abolished by 6-OHDA treatment, supporting the role of sympathetic innervation in SCN-regulated BAT activation.

Regarding the ADRB3 antagonist (SR59230A), we agree that corresponding OCR data would further strengthen the conclusions. However, due to the lengthy 12-week experimental timeline required for TRF-STE and SCN manipulation, our current cohort was not sufficient to perform additional mitochondrial isolation and functional assays post-SR treatment. If deemed essential, we would be happy to undertake this experiment and respectfully request a three-month extension to generate a new mouse cohort.

Importantly, as an alternative readout, we show that SR59230A treatment abolished both (1) the thermogenic advantage of SCN lesioned mice (S3H and S3I Fig), and (2) the upregulation of *S100b* and *p21* expression (Fig 5I), suggesting that β3-adrenergic signaling is functionally required for both thermogenic output and S100B-mediated proliferative responses. We hope these complementary data sufficiently address the reviewer's concerns.

*- The authors should clarify whether S100B is expressed and modified in other cell types, based on the snRNAseq shown in Fig. 3J. What is the contribution of the other cell types? Similarly, to assert that EdU staining demonstrates preadipocyte proliferation in vivo, the authors should use a preadipocyte-specific marker in addition to EdU staining.*

**Response:**

Thank you for pointing this out. In the revised manuscript, we have clarified that snRNA-seq revealed elevated *S100b* expression specifically in brown adipocytes of SCN-lesioned mice at both ZT4 and ZT16, with no significant changes in other cell types on page 13 lines 18 to 21 (S5A and S5B Fig, S4 Table).

As suggested, we now provide PDGFRα immunostaining (S4G and S4H Fig), showing a significantly higher number of PDGFRα⁺ preadipocytes in SCN-lesioned mice compared to sham controls under both Ad-STE and TRF-STE. This confirms that EdU incorporation largely reflects preadipocyte proliferation on page 12, lines 3-6.

*- The authors should discuss the relevance of changes in adipocyte proliferation in this context, in relation to the use of energy substrates.*

Thank you for this suggestion. We have added a paragraph in the discussion “*The observed increase in preadipocyte proliferation under SCN disruption likely represents an adaptive mechanism to maintain BAT thermogenic capacity during nutrient and thermal stress. Expanded pools of preadipocytes provide a sustained source of brown adipocytes, thereby enhancing tissue plasticity. Importantly, proliferative activity is coupled to a metabolic switch: while sham mice under TRF-STE suppress proliferation and favor lipid conservation, SCN-lesioned mice maintain proliferation alongside a shift toward glucose utilization. This coupling suggests that BAT expansion under SCN dysfunction is fueled by carbohydrate substrates, which provide a readily accessible energy source for proliferating cells and thermogenesis. Thus, proliferation not only sustains BAT mass but also integrates with substrate reallocation to preserve thermogenic output during energy stress, where lipid mobilization is impaired.*” on page 18, lines from 24 to 35.

*- The graphical abstract need to be improved, in particular by adding TRF and ZT.*

**Response:**

Thank you for this suggestion. We added description: *Working model illustrating the SCN-ADRB3-S100B axis in BAT during TRF conducted at ZT16-ZT20 in a subthermoneutral environment, the SCN regulates BAT thermogenic plasticity via SNS output. SCN lesioning enhances ADRB3 signaling and S100B expression, which together form a positive feedback loop that amplifies β3-adrenergic sensitivity, promotes preadipocyte proliferation, suppresses senescence, and sustains glucose-driven thermogenesis* in Fig 6V (Fig 7H in revised version) caption.

*Reviewer #3: Zeng et al. provide a well-written and well-presented series of studies which support the conclusion that the SCN affects BAT themogenic plasticity via the SNS and ADRB3-S100B axis.*

*I have no major comments, but several minor ones. I present the minor comments mostly in order in which they appear in the manuscript.*

*Results.*

*Section 1, paragraph 2. "To account for these compositional differences…" This section is written as if the variable of fat or lean mass is what is compared to EE. However, the graph indicates that it is not body composition (fat or lean), but whole body weight that is tested against EE. This analysis allows one to conclude that SCNx reduces EE relative to body weight. Given the increase in body weight variability in the SCNx group (compared to WT), this test is appropriate. However, the text description of why the test was done (to look at composition differences) does not appear accurate. The final sentence of this paragraph should be edited to capture this nuance.*

*Discussion section. Related to the comment above, the discussion indicates that GLM accounted for differences in body composition. While that may be true, the graph in the figure suggests otherwise.*

**Response:**

We appreciate this clarification. We have revised the sentence “*To determine whether SCN lesioning alters EE independently of body mass, we applied a General Linear Model (GLM) with body weight as a covariate.*” On page 5, Fig 1K and S1H.

We also deleted the Discussion about GLM, which accounted for differences in body composition.

*Section 1, paragraph 3. "HFD-fed mice still experienced significant hypothermia". This description is a bit too vague for a results section - mainly because in the 1st paragraph of this section, the authors do a nice job of describing the BAT, core, and tail temperature. The sentence describing Fig. 1L should be edited so that the reader is aware that this is only referring to core temperature. This edit doesn't change the authors' conclusions, merely provides a more accurate and full description of the data.*

**Response:**

Thank you for pointing this out. Revised as: “*HFD-fed mice still exhibited significantly lower core body temperature during TRF-STE (Fig 1L).*”

*Section 3, paragraph 2. "…abolished the elevated thermogenic response observed in SCN lesioned mice…". While I agree with the authors that this is the likely interpretation, the graph/results are not comparing "SCNx with 6-OHDA" to "SCNx without 6-OHDA". The results would be more accurately stated as "6-OHDA treatment produces thermogenic responses that are indistinguishable between sham and SCN lesioned animals" (or similar). This edit doesn't change the authors' conclusions, merely provides a more accurate presentation of the data.*

**Response:**

Thank you for pointing this out. Revised as: “*Under TRF-STE conditions, 6-OHDA treatment resulted in thermogenic responses that were indistinguishable between sham and SCN-lesioned mice, indicating that intact sympathetic innervation is required for SCN-dependent BAT activation (Figs 3E, S3F and S3G).*”

*Section 3, paragraph 2. Similar comment as above, but now in reference to the ADRB3 antagonist.*

**Response:**

Thank you for pointing this out. Revised as: “*Following SR59230A administration, sham and SCN-lesioned mice exhibited comparable core and BAT temperatures under TRF-STE (Figs 3F, S3H and S3I), supporting the conclusion that β3-adrenergic signaling is a critical mediator of SCN-dependent thermogenic regulation.*”

*Section 4, first sentence. "molecular" likely is meant to be "mechanism" or "molecular mechanism"*

**Response:**

Thank you for pointing this out. Corrected to “*molecular mechanism.*”

*Section 4, second to last paragraph, last sentence. "…indicating that SCN activity modulates BAT…". The word "indicating" is too strong for the experiments described since the authors have not yet presented the data that would best support the role of the SCN yet. I would advise changing this word to "suggesting" or "supporting the idea".*

**Response:**

Thank you for pointing this out. Revised as “*suggesting*.”

*Section 5, third paragraph. "This intervention ____ the expression levels of S100b…" The authors are missing a word between "intervention" and "the expression levels".*

**Response:**

Thank you for pointing this out. Revised as: “*This intervention eliminated the expression differences in S100b, Ccnd1 and Cdkn1a between SCN-lesioned and sham mice (Fig 5I), indicating that ADRB3 signaling modulates the S100B-mediated balance between proliferation and senescence.*”

*Section 6. Please describe Fig. 6V in the results section and/or provide a narrative in the discussion.*

**Response:**

Thank you for pointing this out. We added the following description in the Discussion: “*Here, we demonstrate that the SCN, the master circadian pacemaker, coordinates metabolic flexibility in BAT by integrating nutrient signals with sympathetic outflow to regulate substrate utilization and thermogenesis. Specifically, under TRF-STE, intact SCN activity suppresses BAT proliferation and promotes senescence, whereas SCN disruption elevates S100B expression mimicking HFD effect in the absence of lipids, promoting preadipocyte proliferation, and enables BAT to sustain thermogenesis through a glucose-driven program. Importantly, the positive feedback loop between S100B and ADRB3 sustains thermogenic output in the absence of lipid supply (Revised 7H).”*

*Discussion Section.*

*The authors note in Results, section 5, that the present results contrast with prior publications, which find increased serum S100B with obesity and neuroinflammation. Can the authors comment on the possible discrepancy between prior studies and theirs?*

**Response:**

Thank you for raising this important point. Indeed, prior studies in humans have consistently reported elevated serum S100B in association with obesity, neuroinflammation, or metabolic syndrome (e.g., Michetti et al., 2024; Steiner et al., 2010). By contrast, in our study, serum S100B remained unchanged when comparing SCN-lesioned and sham mice. In contrast, local BAT S100B expression was markedly altered under different nutritional states and SCN disruption. We believe this discrepancy arises from tissue specificity: in obesity and neuroinflammatory contexts, serum S100B likely reflects contributions from multiple sources, including brain astrocytes and adipose depots, whereas in our controlled model, the SCN-BAT axis primarily drives local BAT-specific regulation of S100B, without systemic spillover into circulation. This suggests that serum S100B may serve as a biomarker of systemic stress in chronic disease. At the same time, local BAT S100B functions as a nutrient-sensitive effector of thermogenic plasticity under acute energetic stress.

We have clarified this distinction in the revised Discussion: *“Thus, S100B acts not as a direct cold sensor but as a nutrient-sensitive amplifier of sympathetic input. This dual regulation reconciles our findings with previous reports that serum S100B is elevated in obesity and neuroinflammation. Whereas those studies reflected systemic or central changes, our data indicate that in BAT, S100B functions locally as an amplifier of β3-adrenergic signaling and a regulator of cellular plasticity.*” on page 18 lines 15 to line 19.

*Other.*

*There is no mention as to whether the mice used were males, females, or a mix of each. Please clearly indicate the sex used at each mention of mice in the method section (e.g., whenever the age of the mice is mentioned, so should the sex). If using of a male-only study design, justify the rationale and add "in male mice" to paper title.*

**Response:**

We apologize for this omission. All experiments in this study were conducted in male C57BL/6J mice. We chose a male-only design to avoid potential confounding effects of estrous cycle-associated fluctuations in body temperature, activity, and adipose tissue metabolism, which could complicate interpretation of SCN-dependent thermogenic regulation. This approach is consistent with prior circadian and metabolic physiology studies, where sex-specific hormonal variation may mask subtle neural-metabolic interactions. In the revised manuscript, we have:

1. Clearly indicate the sex of mice in the Methods (e.g., “8-week-old male C57BL/6J mice”).
2. Updated the title to include the qualifier: *“*Suprachiasmatic nucleus regulation of brown fat thermogenesis via the ADRB3-S100B axis in male mice.*”*
