# Supplementary material for: The suprachiasmatic nucleus regulates brown fat thermogenesis in male mice through an adrenergic receptor ADRB3-S100B signaling pathway
Source: PLoS Biol. 2025 Dec 4;23(12):e3003534. doi: 10.1371/journal.pbio.3003534 (PMC12688110; doi:10.1371/journal.pbio.3003534)
Supplement: Attachment — Submitted filename: Response to reveiwers2.doc [file pbio.3003534.s016.doc]

Response to Reviewers
Manuscript ID: PBIOLOGY-D-25-01865R2
Title: “The suprachiasmatic nucleus regulates brown fat thermogenesis in male mice through an adrenergic receptor ADRB3-S100B signaling pathway”

**Point-to-point response**

*Reviewer #1
I consider the manuscript with the current changes as acceptable.*
**Response:** We thank the reviewer for the positive evaluation.

*Reviewer #2: The quality of the main figures in this PDF version was suboptimal, most likely due to a formatting issue during the upload process. Nevertheless, the authors have conducted substantial additional work that considerably enhances the overall quality of the manuscript.*

*In response to my previous comments, the authors have provided the requested information in the figure legends, quantified adipocyte size, performed additional mitochondrial respiration experiments, carried out complementary analyses on the sn-RNAseq data, and added new immunostaining experiments that further support the proliferative nature of preadipocytes. They have also addressed the discussion points raised in the previous round of review and modified the graphical abstract accordingly. Taken together, these revisions and additions have greatly improved the manuscript, and I thank the authors for their efforts and clarifications.*

**Response:** First of all, thank you for your positive assessment of this revision. We sincerely apologize for the poor quality of the figures in the system-generated PDF; we have uploaded additional high-resolution images that meet the journal’s requirements.

*With regard to the comments made by the other reviewers, particularly those concerning the control animals shown in Figure 1, I wonder whether these animals originate from independent experiments. If that is the case, this information should be explicitly mentioned in the text with an appropriate reference, and the corresponding curve should be represented as a dashed line in the figure to indicate this distinction.*

*Overall, the authors' responses are well thought out and have significantly strengthened the manuscript through the inclusion of new controls, additional experiments, and an improved discussion section. It is also important to emphasize the broad scope of this study, which includes an impressive range of models, experiments, and results. For all these reasons, I recommend the manuscript for publication.*

**Response:** Thank you for your suggestion. The control data in Fig 1 were derived from an independent experiment; we have now added the data source and the appropriate citation in the main text and figure legend of Fig 1(Page 5, lines 10–11), and changed the corresponding curve to a dashed line to indicate this distinction.

*Reviewer #3*

*Comment: The author addressed all my comments.*

**Response:** We thank the reviewer for confirming that all concerns have been resolved.
